# Guidance for overviews of reviews continues to accumulate, but important challenges remain: a scoping review

**DOI:** 10.1186/s13643-020-01509-0

**Published:** 2020-11-04

**Authors:** Michelle Gates, Allison Gates, Samantha Guitard, Michelle Pollock, Lisa Hartling

**Affiliations:** 1 Alberta Research Centre for Health Evidence, Department of Pediatrics, University of Alberta, 4-486C Edmonton Clinic Health Academy, 11405-87 Avenue NW, Edmonton, AB T6G 1C9 Canada; 2 Alberta Research Centre for Health Evidence, Department of Pediatrics, University of Alberta, 4-482C Edmonton Clinic Health Academy, 11405-87 Avenue NW, Edmonton, AB T6G 1C9 Canada; 3 Alberta Research Centre for Health Evidence, Department of Pediatrics, University of Alberta, 4-488C Edmonton Clinic Health Academy, 11405-87 Avenue NW, Edmonton, AB T6G 1C9 Canada; 4 Health Technology Assessment Unit, Institute of Health Economics, 1200 10405 Jasper Avenue, Edmonton, AB T5J 3N4 Canada; 5 Alberta Research Centre for Health Evidence, Department of Pediatrics, University of Alberta, 4-472 Edmonton Clinic Health Academy, 11405-87 Avenue NW, Edmonton, AB T6G 1C9 Canada

**Keywords:** Overview of reviews, Umbrella review, Metareview, Systematic reviews, Knowledge synthesis, Evidence synthesis, Evidence-based medicine, Scoping review, Metasummary

## Abstract

**Background:**

Overviews of reviews (overviews) provide an invaluable resource for healthcare decision-making by combining large volumes of systematic review (SR) data into a single synthesis. The production of high-quality overviews hinges on the availability of practical evidence-based guidance for conduct and reporting.

**Objectives:**

Within the broad purpose of informing the development of a reporting guideline for overviews, we aimed to provide an up-to-date map of existing guidance related to the conduct of overviews, and to identify common challenges that authors face when undertaking overviews.

**Methods:**

We updated a scoping review published in 2016 using the search methods that had produced the highest yield: ongoing reference tracking (2014 to March 2020 in PubMed, Scopus, and Google Scholar), hand-searching conference proceedings and websites, and contacting authors of published overviews. Using a qualitative meta-summary approach, one reviewer extracted, organized, and summarized the guidance and challenges presented within the included documents. A second reviewer verified the data and synthesis.

**Results:**

We located 28 new guidance documents, for a total of 77 documents produced by 34 research groups. The new guidance helps to resolve some earlier identified challenges in the production of overviews. Important developments include strengthened guidance on handling primary study overlap at the study selection and analysis stages. Despite marked progress, several areas continue to be hampered by inconsistent or lacking guidance. There is ongoing debate about whether, when, and how supplemental primary studies should be included in overviews. Guidance remains scant on how to extract and use appraisals of quality of the primary studies within the included SRs and how to adapt GRADE methodology to overviews. The challenges that overview authors face are often related to the above-described steps in the process where evidence-based guidance is lacking or conflicting.

**Conclusion:**

The rising popularity of overviews has been accompanied by a steady accumulation of new, and sometimes conflicting, guidance. While recent guidance has helped to address some of the challenges that overview authors face, areas of uncertainty remain. Practical tools supported by empirical evidence are needed to assist authors with the many methodological decision points that are encountered in the production of overviews.

**Supplementary Information:**

The online version contains supplementary material available at 10.1186/s13643-020-01509-0.

## Background

By systematically identifying and synthesizing all available evidence for a particular research question, systematic reviews are considered foundational to evidence-based healthcare [1]. It is estimated that 8000 systematic reviews were published in 2014 [2], more than three times the yearly publication rate recorded 10 years earlier [3]. Around the turn of the century overviews of reviews, which compile data from multiple systematic reviews, emerged to deal with the growing volume of published systematic reviews [4, 5]. By taking advantage of existing syntheses, overviews of reviews can create efficiencies [6] and answer broader research questions [7].

Many of the methods used to undertake systematic reviews are suitable for overviews of reviews, but their conduct also presents unique methodological challenges [7, 8]. Many methods to conduct the various stages of overviews of reviews have been suggested; however, much of the guidance is inconsistent, and evidence-based reporting guidance is lacking [9]. The relative lack of evidence and consistency in recommendations may underpin the inadequate and inconsistent conduct and reporting of overviews to date [4, 5, 10]. As the science of overviews of reviews continues to develop, authors will need to keep up-to-date with the latest methods research and reporting guidelines [11].

In an effort to collate available guidance for overview conduct and to inform future evaluations aimed at advancing the science of overviews, in 2016 our team published a scoping review of guidance documents for researchers conducting overviews of reviews of healthcare interventions [12]. In addition, we completed a methodological systematic review examining the quality of reporting of a sample of overviews of reviews of healthcare interventions published from 2012 to 2016 [13], updating earlier work by our team [4, 5]. To address the gap in guidance for reporting, in 2017 we registered our intent to develop an evidence-based and consensus-based reporting guideline for overviews of reviews (Preferred Reporting Items for Overviews of Reviews (PRIOR)) with the Equator Network [14]. We used evidence from our aforementioned reviews to inform the preliminary list of items for PRIOR [9, 15]. In order to ensure that the items were informed by the most up-to-date available guidance, herein we have updated our existing scoping review to include new guidance documents that have become available in the past four years [14]. In the future, this work may be extended to develop minimum standards of methodological conduct for overviews.

The aims of this updated scoping review were to (1) locate, access, compile, and map documents that provide explicit methodological guidance for conducting overviews of reviews; (2) identify and describe areas where guidance for conducting overviews of reviews is clear and consistent, and areas where guidance is conflicting or missing; and (3) document common challenges involved in conducting overviews of reviews and determine whether existing guidance can help researchers overcome these challenges [12].

## Methods

We updated the scoping review published by Pollock et al. in 2016 [12]. In doing so, we followed very similar methodology to the original scoping review, with the exception of alterations to the search to increase feasibility. We adhered to the methodological framework described by Arksey and O’Malley [15] and refined by Levac et al. [16]. We reported our intent to update the 2016 review in our protocol for the development of the PRIOR guideline [9]. Reporting adheres to the Preferred Reporting Items for Systematic Reviews and Meta-Analyses extension for scoping reviews (PRISMA-ScR; checklist in Additional file 1) [17].

### Eligibility criteria

We included documents produced in any format, language, or year that either (a) provided explicit guidance related to the context or process of any aspect of conducting overviews of reviews examining the efficacy, effectiveness, and/or safety of healthcare interventions, or (b) described an author team’s experience in conducting overviews of reviews of healthcare interventions. When selecting documents for inclusion, we used a pre-established definition of overviews of reviews (Table 1). This definition was recently published by Cochrane [18], and was informed by Pollock’s 2016 scoping review [12]. We excluded existing overviews, documents that were not about overviews of health interventions (including those reporting on different types of overviews, e.g., diagnostic or etiology), and those that were not intended as methods guidance. We excluded guidance for the reporting of overviews, because we viewed reporting as a distinct concept that is informed by guidance about overview conduct. We also excluded guidance documents that had been updated and superseded since the 2016 review, and conference abstracts for which a full version of the document was available that provided additional information.

**Table 1:** Definition of “overview of reviews” [18]

An overview of reviews:	
1. Contains a clearly formulated objective designed to answer a specific research question, typically about a healthcare intervention. 2. Intends to search for and include only systematic reviews (with or without meta-analyses). 3. Uses explicit and reproducible methods to identify multiple systematic reviews that meet the overview of reviews’ inclusion criteria and assess the quality/risk of bias of these systematic reviews. 4. Intends to collect, analyze, and present the following data from included systematic reviews: descriptive characteristics of the systematic reviews and their included primary studies; risk of bias of primary studies; quantitative outcome data; and certainty of evidence for pre-defined, clinical important outcomes. 5. Discusses findings as they relate to the purpose, objective(s), and specific research question(s) of the overview of reviews, including: a summary of main results, overall completeness and applicability of evidence, quality of evidence, potential biases in the overview process, and agreements and/or disagreements with other studies and/or reviews.	

### Searches

We conducted an iterative and extensive search to ensure breadth and comprehensiveness of coverage [15, 16, 19, 20], with the assistance of a research librarian (Additional file 2). The searches for the original scoping review covered the period from January 2010 to December 2013 (for databases), and to 2015 for snowballing and other searches (described in detail within the publication) [12]. The review included a search of online databases (Medline and Embase via Ovid, DARE and the Cochrane Methods Study Database via the Cochrane Library, Medline via Web of Science), reference tracking in Scopus and PubMed, article alerts from Google Scholar and Web of Science, hand-searching 26 websites and conference proceedings for three conferences, and contacting producers of overviews [12]. Based on the experience of the lead author of the original scoping review, for feasibility we included the search methods with the highest yield in this update (i.e., reference tracking and hand-searching, while eliminating the term-based database search). In the previous scoping review, 71% of included documents were located using these methods [12]. This allowed us to complete the scoping review on an expedited timeline, while including the most recent guidance as it became available.

On 7 March 2019, we conducted an iterative reference tracking (“snowballing”) search [19, 20]. We used 46 target articles, including all published articles and abstracts cited in the 2016 scoping review [21], as well as other recent relevant articles known to the research team. For each target article, we searched for “citing” references in Google Scholar and Scopus and for “similar articles” in PubMed from 1 January 2014 to present. Following the initial searches, the “citing” references search in Scopus and “similar articles” search in PubMed were turned into monthly e-mail alerts. We augmented the reference tracking with searches of Google Scholar, using terms that are commonly used to describe overviews, such as “overview of reviews,” “umbrella review,” and “review of systematic reviews.” The initial search was run on 1 March 2019 and restricted to documents available since 2014, corresponding to the end date of the previous database searches. The search was then turned into an e-mail alert. The last date searched for all electronic sources was 1 March 2020.

In addition to the electronic searches, on 6–12 February 2019, we (MG, AG, SG) hand-searched the websites of 59 organizations (33 additional since the original review) that had conducted at least one overview of reviews and of major evidence synthesis centres (Additional file 2). We also hand-searched the conference proceedings from four international conferences: the International Cochrane Colloquium (2015–present), Health Technology Assessment International (2017–present), the Canadian Agency for Drugs and Technologies in Health Symposium (2015–present), and the Global Evidence Summit (added in this iteration; 2017). These searches were updated on 3–5 February 2020. We also reviewed the reference lists of newly included documents.

On 26 February 2019, we e-mailed content experts to inquire about additional relevant studies. We contacted the primary or senior authors of a random sample of 100 overviews published between 2012 and 2016; this was the same sample used in our team’s aforementioned methodological systematic review examining the quality of reporting of overviews [13]. We also contacted 22 managing editors of Cochrane Review Groups that had published at least one overview of reviews. If we did not receive a reply, we sent a second e-mail on 27 March 2019 before ceasing contact. We received responses from 51 authors and 19 managing editors.

### Document selection

Two independent reviewers (MG and SG) screened the titles and abstracts of documents retrieved by the electronic searches in Excel. We retrieved the full texts of all potentially relevant documents identified by either of the reviewers. One reviewer (MG) scanned the websites and retrieved the full texts of potentially relevant documents, while another (SG) retrieved the full texts of documents recommended by content experts. The two reviewers independently scanned the conference proceedings and retrieved the full texts for all believed to be potentially relevant by either reviewer. Both reviewers independently reviewed all full texts and agreed on those that were ultimately included, with disagreements resolved through discussion or the involvement of a third reviewer (AG).

### Data extraction and synthesis

Either of three reviewers (MG, SG, or AG) read the included documents and extracted and synthesized relevant text using a qualitative meta-summary approach [22, 23]. This is a quantitatively oriented approach to aggregating qualitative findings that includes extracting, editing, grouping, formatting, and presenting findings across groups of related documents [22–24]. The first reviewer read each of the documents and highlighted text providing guidance on any stage of the overview of reviews process and/or describing challenges in undertaking an overview of reviews. Each full text was then read by a second reviewer who confirmed and/or edited the highlighting and extracted relevant text to a data extraction file in Microsoft Excel. The first reviewer then verified the data extraction and corrected errors and/or omissions. Finally, one reviewer edited the guidance and challenges extracted from all documents to ensure that they were presented in a way that was accessible to readers while preserving their underlying content and meaning [24].

Next, we followed a two-stage approach to group similar findings. We began by grouping all documents produced by the same research group to avoid giving extra weight to statements included in multiple documents from the same group [24]. Then, we grouped statements across research groups by stage of the overview process [24] in a way that aligned with the 2016 version of this scoping review [12]. These stages included items related to the context of conducting an overview (e.g., types of questions that may be answered, items to consider, author team composition, and target audience), and items related to the process of conducting overviews (e.g., protocol development, search, selection, data extraction, quality appraisal, synthesis, assessing certainty, developing conclusions, updating the overview). Finally, within each group, we developed a refined list of guidance statements by editing the findings to organize topically similar statements together and to eliminate redundancies, while conserving ambiguities.

Finally, we developed a narrative summary of the extracted guidance by stage of the overview process, and challenges. For the guidance statements, we also calculated the frequency and intensity effect sizes [23, 24]. We calculated frequency effect sizes by dividing the number of research groups contributing guidance on a topic area by the total number of research groups. We calculated intensity effect sizes by dividing the number of topic areas addressed by each research group by the total number of topic areas.

## Results

**Fig. 1:**
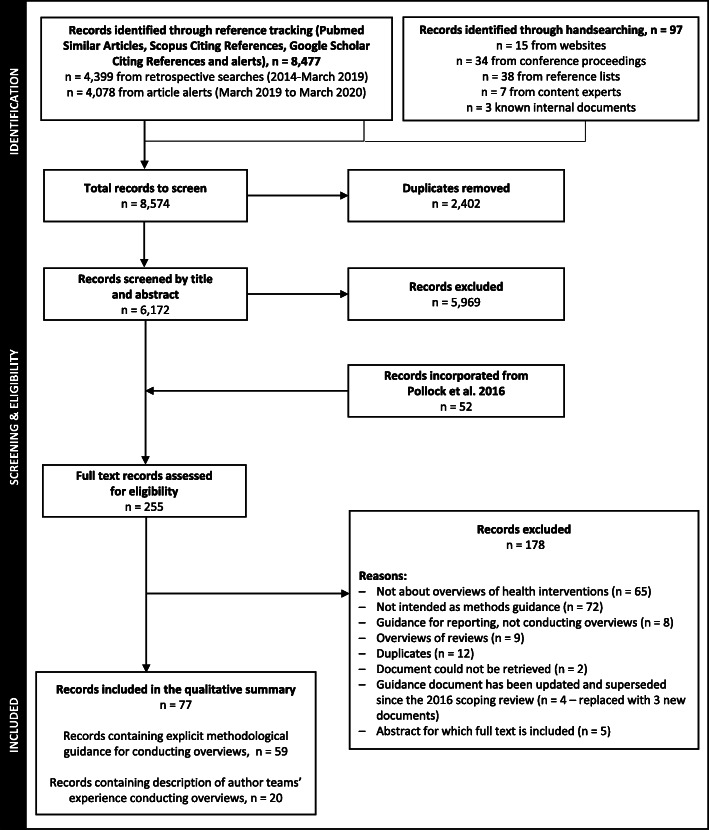
Flow diagram of document selection

The searches retrieved 6173 records that were screened by title and abstract, after which 5969 were excluded. After incorporating the 52 documents included in the original scoping review (41 reporting guidance and 11 reporting experiences), we assessed the full text of 254 and included 28 new documents (Fig. 1; studies excluded at full-text review in Additional file 3). Four guidance documents that were included in the original scoping review were excluded and replaced with three new documents that updated and superseded the previous guidance. There are now 77 documents produced by 34 research groups included in this scoping review, which were published (or became available) between 2009 and 2020. These documents, with their abbreviations (used within the results and tables), are listed in Additional file 4, and labelled as “A1,” “A2”… throughout the review.

Of these, 59 documents (21 new since the previous iteration) produced by 24 research groups provided explicit methods guidance for conducting overviews of reviews, and 20 documents (9 new) produced by 16 research groups described author teams’ experience conducting published overviews of reviews (Table 2). Two documents reported both methodological guidance and on authors’ experiences conducting overviews (A17, A47). There were 30 (39%) conference presentations, 27 (35%) journal articles, 9 (12%) internal documents, 5 (6%) book sections or chapters, 2 (3%) websites, and one each of editorials, dissertations, case reports, and interview transcripts. In the sections that follow, we summarize the guidance and challenges provided in these documents; this includes all documents located to date (i.e., incorporating both those located in the Pollock 2016 review [12] and our update).

**Table 2:** Characteristics of the included documents (77 documents produced by 34 research groups/author teams)

Author team or research group	Documents containing explicit methodological guidance for conducting overviews (59 documents produced by 24 research groups)			Documents that describe authors teams’ experience conducting published overviews (20 documents produced by 16 research groups)		
	*N* documents (reference #–Additional file 4)	Years of publication	Document formats	*N* documents (reference #–Additional file 4)	Years of publication	Document formats
Alberta Research Centre for Health Evidence (ARCHE)	3 (A1-A3)	2017–2019	2 journal articles, 1 associated thesis	1 (A60)	2019	1 journal article
Belgian Health Care Knowledge Centre (KCE)	1 (A4)	2016	1 journal article	–	–	–
Central South University (CSU)	1 (A5)	2020	1 journal article	–	–	–
Cochrane Child Health Field (CHF)	11 (A6–A16)	2010–2015	8 conference presentations, 2 internal documents, 1 journal article	2 (A61–A62)	2011–2013	1 journal article, 1 conference presentation
Cochrane Chile (CChile)	1 (A17)	2019	1 conference presentation	1 (A17)	2019	1 conference presentation
Cochrane Comparing Multiple Interventions Methods Group (CMIMG)	19^a^ (A6, A13, A14, A18-A36)	2008–2019	8 conference presentations, 6 internal documents, 2 websites, 1 handbook chapter, 1 journal article, 1 interview transcript	–	–	–
Cochrane Consumers and Communication Review Group (CCRG)	–	–	–	1 (A63)	2009	1 journal article
Cochrane Effective Practice and Organization of Care Group (EPOC)	1 (A37)	2011	1 conference presentation	3 (A64–A66)	2011–2019	3 conference presentations
Cochrane Musculoskeletal Group (CM)	–	–	–	1 (A67)	2011	1 conference presentation
Cochrane Public Health Group (CPHG)	1 (A38)	2014	1 journal article	–	–	–
Cochrane Stroke Group (CSG)	–	–	–	1 (A68)	2015	1 conference presentation
Duke University (DukeU)	1 (A39)	2012	1 journal article	–	–	–
Dutch Cochrane Centre (DCC)	–	–	–	1 (A69)	2009	1 conference presentation
Evidence for Policy and Practice Information and Co-ordinating Centre (EPPI)	2 (A40–A41)	2015	1 journal article, 1 conference presentation	–	–	–
Glasgow Caledonian University (GCU)	1 (A42)	2019	1 journal article	2 (A70–A71)	2016–2017	2 journal articles
Harvard University and the Cyprus University of Technology (HarvU)	1 (A43)	2019	1 journal article	–	–	–
Joanna Briggs Institute Umbrella Reviews Methodology Group (JBI)	3 (A44–A46)	2013-2017	1 journal article, 1 internal document, 1 book chapter	–	–	–
King’s College London (KCL)	1 (A47)	2018	1 journal article	1 (A47)	2018	1 journal article
Ludwig Boltzmann Institute for Health Technology Assessment (LBI)	–	–	–	1 (A72)	2015	1 journal article
Northeast Institute of Evidence Synthesis and Translation at Rutgers School of Nursing (NEST)	1 (A48)	2016	1 book chapter	–	–	–
Norwegian Knowledge Centre for the Health Services (NOKC)	1 (A49)	2013	1 book chapter	–	–	–
Pontifical Xavierian University (PXU)	–	–	–	1 (A73)	2011	1 conference presentation
Robinson Research Institute, University of Adelaide (RRI)	–	–	–	1 (A74)	2016	1 conference presentation
Sapienza University of Rome (SUR)	1 (A50)	2016	1 book section	–	–	–
Trinity College Dublin (TCD)	1 (A51)	2011	1 journal article	–	–	–
University of Auckland (UAuck)	–	–	–	1 (A75)	2019	1 conference presentation
University of Birmingham (UBirm)	1 (A52)	2012	1 journal article	–	–	–
University of Calgary (UCalg)	–	–	–	1 (A76)	2017	1 case report
University of Connecticut (UConn)	2 (A53–54)	2019	2 journal articles	–	–	–
University of Cyprus (UCyp)	1 (A55)	2020	1 journal article	–	–	–
University of Dundee (UDun)	–	–	–	1 (A77)	2004	1 journal article
University of Oxford (UOx)	1 (A56)	2017	1 journal article	–	–	–
Western Journal of Nursing Research (WJNR)	1 (A57)	2014	1 editorial	–	–	–
Witten/Herdecke University (WHU)	2 (A58–59)	2014	2 journal articles	–	–	–

^a^Three documents (conference presentations) not counted because they were produced by authors also affiliated with CMIMG (duplicates)

### Guidance for conducting overviews of reviews

Since the previous version of this review [12], 21 new guidance documents related to the context or conduct of overviews of reviews became available (ARCHE: A1, A2, A3; KCE: A4; CSU: A5; CChile: A17; CMIMG: A23, A24, A25, A27, A28; GCU: A42; HarvU: A43; JBI: A44; KCL: A47; NEST: A49; SUR: A50; UConn: A53, A54; UCyp: A55; UOx: A56). The new documents contained guidance relating to all of the 15 topic areas included in the previous review. We also added two new topic areas: developing and registering the overview of reviews protocol; and updating the overview of reviews. Table 3 shows a map of the guidance provided by these documents. The number of topics addressed by each research group was median (range) 8 (1 to 17); three groups addressed ≥ 15 of the topic areas in their guidance documents (CMIMG, JBI, SUR). The number of groups reporting on each topic area was median (range) 11 (3 to 21). Within the following sections, for each stage of the overview process, we provide a narrative summary of the guidance available.

**Table 3:** Map of available guidance for the conduct of overviews of reviews

Topic	ARCHE	KCE	CSU	CChile	CHF	CMIMG	EPOC	CPHG	DukeU	EPPI	GCU	HarvU	JBI	KCL	NEST	NOKC	SUR	TCD	UBirm	UConn	UCyp	UOxf	WJNR	WHU	Frequency effect size (of 24)
Guidance related to the context of conducting an overview of reviews																									
What types of questions can be answered using the overview format?					✓	✓			✓		✓		✓			✓	✓		✓			✓			9
Deciding between conducting an overview and a systematic review		✓				✓				✓			✓				✓			✓		✓			7
Items to consider before deciding to conduct an overview			✓		✓	✓				✓	✓	✓	✓	✓			✓	✓		✓		✓	✓	✓	14
Author team composition and roles					✓	✓			✓				✓				✓	✓		✓			✓		8
Target audience of the overview					✓	✓	✓	✓	✓	✓	✓		✓				✓	✓			✓		✓	✓	13
Guidance related to the process of conducting an overview of reviews																									
Developing and registering an overview protocol						✓					✓	✓	✓	✓			✓			✓	✓				8
Specifying the scope					✓	✓	✓	✓	✓	✓			✓		✓	✓	✓	✓		✓			✓		13
Searching for systematic reviews (and potentially primary studies)		✓	✓		✓	✓	✓	✓	✓	✓			✓		✓	✓	✓	✓	✓	✓	✓		✓	✓	18
Selecting systematic reviews for inclusion (and potentially primary studies)	✓	✓	✓		✓	✓	✓	✓	✓	✓		✓	✓		✓	✓	✓	✓		✓	✓	✓	✓	✓	20
Should an overview include non-Cochrane systematic reviews?					✓	✓				✓			✓												4
Assessing the quality of included systematic reviews	✓	✓	✓		✓	✓	✓	✓	✓	✓		✓	✓	✓	✓	✓	✓	✓	✓	✓	✓		✓		20
Collecting and presenting data on descriptive characteristics of the included systematic reviews		✓			✓	✓	✓		✓				✓		✓		✓	✓		✓			✓		11
Collecting and presenting data on the quality of primary studies contained within the systematic reviews		✓			✓	✓			✓	✓		✓	✓			✓	✓						✓		10
Collecting, analyzing, and presenting outcome data		✓	✓	✓	✓	✓	✓		✓	✓	✓	✓	✓	✓	✓	✓	✓	✓	✓	✓	✓		✓	✓	21
Assessing the certainty/quality of the body of evidence		✓	✓		✓	✓			✓			✓	✓		✓	✓	✓	✓			✓			✓	13
Interpreting outcome data and drawing conclusions		✓				✓	✓		✓			✓	✓										✓	✓	8
Updating the overview						✓					✓						✓								3
Intensity effect size (of 17)	2	9	6	1	13	17	9	6	12	10	6	8	16	4	7	8	15	10	4	10	7	4	11	7	

#### Types of questions that can be answered using the overview of reviews format

There is limited guidance on the types of questions that can be answered using the overview of reviews format (CMIMG: A27, SUR: A50), with most (*n* = 7) groups citing CMIMG guidance in their documents (CHF: A12, DukeU: A39, GCU: A42, JBI: A44, NCHS: A49, TCD: A51, UBirm: A52). Cochrane indicates that overviews of reviews can be used to summarize information on “different interventions for the same condition; different outcomes for the same intervention in the same condition; the same intervention for different conditions or populations; adverse effects across multiple conditions” (CMIMG: A27). Chapter 5 of Biondi-Zoccai’s book “Umbrella Reviews” cites similar questions, with the addition of summarizing information on the “adverse effects of multiple interventions for a specific condition” (SUR: A50).

#### Choosing between conducting an overview of reviews and a systematic review

The available guidance states that overviews of reviews may be considered when the purpose is to map, synthesize, or explore discrepancies in the available systematic review evidence (JBI: A44, UOx: A56, SUR: A50). Overviews of reviews might be most appropriate when the scope of the research question is broad (CMIMG: A27, EPPI: A40) and an expedited approach is needed (KCE: A4, CMIMG: A27, EPPI: A40). A pre-requisite to performing an overview of reviews is the availability of multiple high quality, up-to-date systematic reviews covering all interventions of interest (KCE: A4, CMIMG: A27, JBI: A44, UOx: A56, UConn: A53). Overviews of reviews are rarely appropriate for identifying research gaps (UOx: A56), ranking interventions, or making indirect comparisons (CMIMG: A27). Decision tools aimed at assisting authors in deciding between conducting a systematic review and an overview of reviews are available in Ballard 2017 (A56) and from Cochrane (A33).

#### Items to consider before conducting an overview of reviews

Before conducting an overview, several groups recommend first ensuring that the topic is clinically important (CHF: A15, CSU: A5, GCU: A42, KCL: A47). Overviews of reviews might not be the best approach when the field is new or rapidly evolving (EPPI: A40), but can be ideal to explore inconclusive evidence across multiple systematic reviews (CSU: A5, HarvU: A43, KCL: A47). Potential authors should scope the literature to ensure that there are up-to-date, high-quality systematic reviews available on all key interventions (CHF: A15, CSU: A5, CMIMG: A27, JBI: A44, KCL: A47, SUR: A50, UConn: A53, UOx: A56, WJNR: A57, WHU: A59), and that it would make sense to combine these in an overview of reviews (CHF: A12, CMIMG: A27). Authors also need to search for existing overviews of reviews in the production phases to prevent research waste (SUR: A50). Important resource and organizational factors to consider include the software that will be used for data management, a realistic time frame, and the size and composition of the author team (SUR: A50, TCD: A51, UConn: A53).

#### Author team composition and roles

Several groups recommend assembling a multidisciplinary author team, which ideally would include a project coordinator (CHF: A11), methodologist (CHF: A16, CMIMG: A27, JBI: A44, TCD: A51, UConn: A53, WJNR: A57), content expert (e.g., clinician) (CHF: A16, CMIMG: A27, DukeU: A39, SUR: A50, TCD: A51), and relevant stakeholders (e.g., patients, decision-makers) (SUR: A50). An information specialist (CHF: A16, CMIMG: A27, SUR: A50, UConn: A53) and/or statistician (CHF: A16, CMIMG: A27, SUR: A50, UConn: A53) may also be needed. At least two authors should be directly involved in day-to-day operations, because many steps should be verified or performed independently in duplicate (JBI: A44, SUR: A50, UConn: A53). If non-English-language systematic reviews are included, it may be necessary to engage first-language speakers (SUR: A50).

#### Target audience of the overview of reviews

Available guidance indicates that the target audience for the overview of reviews may include clinicians and other healthcare providers (CHF, CMIMG: A27, EPOC: A37, CPHG: A38, TCD: A51, WJNR: A57, WHU: A59), researchers (CMIMG: A27, EPOC: 37, DukeU: A39, WJNR: A57), informed consumers (e.g., patients and caregivers) (CMIMG: A27, WHU: A58), policymakers and other healthcare decision-makers (CHF: A7, CMIMG: A27, EPOC: A37, CPHG: A38, EPPI: A40, GCU:A42, JBI: A44, SUR: A50, WJNR: A57, WHU: A59, UCyp: A55), and funding agencies (CMIMG: A27).

#### Developing and registering an overview of reviews protocol

Guidance documents specify that all pre-planned methods should be developed in collaboration with key stakeholders, and be clearly defined (CMIMG: A27, GCU: A42, JBI: A44, KCL: A47, SUR: A50, UConn: A53, UCyp: A55). The protocol should also delineate the goals of the overview of reviews (GCU: A42), the outcomes and effect measures of interest (CMIMG: A27), and the knowledge translation strategy (GCU: A42). Several guidance documents indicate that the protocol should be peer-reviewed and/or published (JBI: A44, KCL: A47, UConn: 53, UCyp: A55), and most recommend that it be registered in an open-access database (HarvU: A43, JBI: A44, KCL: A47, SUR: A50, UConn: A53, UCyp: A55).

#### Specifying the scope of the overview of reviews

Several groups indicate that the scope should be specific and pre-defined based on elements of the populations, interventions, comparators, and outcomes of interest (CMIMG: A27, EPOC: A37, CPHG: A38, JBI: A44, NOKC: A49, SUR: A50, TCD: A51, WJNR: A57). The scope may be narrow, but is often broad, such that the included systematic reviews could be diverse (CMIMG: A27, CPHG: A38, DukeU: A39, JBI: A44, NEST: A48, SUR: A50). In deciding the scope, authors should be aware that there may be full or partial overlap with the scope of potentially eligible systematic reviews (EPPI: A40). The scope should therefore be determined with time and resource limits in mind (UConn: A53). When there is substantial heterogeneity in the questions posed by individual systematic reviews, it might become necessary to restrict the scope of the overview of reviews (CMIMG: A27).

#### Searching for systematic reviews (and potentially primary studies)

Guidance on search procedures indicates that Cochrane systematic reviews can be retrieved via the Cochrane Database of Systematic Reviews (CHF: A15, KCE: A4, CMIMG: A27, TCD: A51, UConn: A53). To locate non-Cochrane systematic reviews, it is recommended that authors search multiple databases (e.g., Medline, EMBASE) (CHF: A15, KCE: A4, CMIMG: A27, EPOC: A37, JBI: A44, NEST: A48, SUR: A50, UConn: A53, WJNR: A57) and registries (e.g., Epistemonikos, PROSPERO) (CMIMG: A27, KCE: A4, CPHG: A38, JBI: A44, NEST: A48, SUR: A50, TCD: A51, UConn: A53), hand-search relevant sources (e.g., webpages) (KCE: A4, JBI: A44, SUR: A50, TCD: A51), screen reference lists (CMIMG: A27, JBI: A44, TCD: A51), and contact relevant individuals and organizations (CMIMG: A27) to find published and non-commercially published systematic reviews. To improve the precision of database searches, systematic review-specific search terms, MeSH headings, and validated filters should be used (CHF: A15, KCE: A4, CMIMG: A27, EPOC: A37, DukeU: A39, JBI: A44, NEST: A48, SUR: A50, TCD: A51, UConn: A53). Authors may consider having search strategies peer-reviewed prior to implementation (TCD: A51). There is a lack of agreement about imposing restrictions based on publication status or language (CMIMG: A27, JBI: A44, SUR: A50, TCD: A51, UConn: A53). Several groups indicate that imposing a date restriction (e.g., past 10 years; pre-1990) could be appropriate (CPHG: A38, JBI: A44, NEST: A48, SUR: A50, TCD: A51. UBirm: A52). There is debate about whether authors should search for primary studies to fill “gaps” in systematic review evidence or to ensure the up-to-dateness of the overview of reviews (CSU: A5, CMIMG: A27, CPHG: A38, DukeU: A39, EPPI: A40, NOKC: A49, SUR: A50, UCyp: A55, WHU: A59).

#### Selecting systematic reviews for inclusion (and potentially primary studies)

Guidance on selecting systematic reviews (and potentially primary studies) for inclusion indicates the importance of clear pre-defined clinical and methodological criteria (ARCHE, KCE, CHF, CMIMG, CSU, EPOC, CPHG, DukeU, EPPI, HarvU, JBI, NEST, NOKC, SUR, TCD, UConn, UOx, WJNR, WHU, UCyp). Authors need to define “systematic reviews” and/or other types of research syntheses that will be included (CSU: A5, CMIMG: A27, EPOC: A37, HarvU: A43, JBI: A44, SUR: A50, UConn: A53, UCyp: A55). Screening should be a transparent and objective two-stage (titles/abstracts, full texts) process (KCE: A4, JBI: A44, NEST: A48, TCD: A51, UConn: A53), preceded by pilot testing (KCE: A4). The process should be performed independently by at least two reviewers, with a procedure in place to resolve disagreements (KCE: A4, EPOC: A37, JBI: A44, SUR: A50, TCD: A51, UConn: A53). When the scope of the overview of reviews differs from the available systematic reviews, authors may need to assess the relevance of their included primary studies, and include only those that match the overview or reviews’ objective (CHF: A15, CMIMG: A27). Several groups indicate that overview of reviews authors may decide to include only high-quality systematic reviews (CHF: A15, DukeU: A39, EPPI: A40, JBI: A44, NEST: A48, NOKC: A49, SUR: A50, TCD: A51, UConn: A53, UOx: A56, WHU: A58), but this risks introducing bias (EPPI: A40, SUR: A50, UConn: A53, UOx: A56). There is diverse guidance about how best to manage overlapping and/or discordant systematic reviews (ARCHE: A3, CHF: A15, CMIMG: A27, DukeU: A39, SUR: A50, UConn: 54). Authors may decide to include all systematic reviews regardless of overlap, or only include the most recent, most comprehensive, most relevant, or highest quality systematic reviews (ARCHE: A3, CHF: A15, CMIMG: A27, DukeU: A39, SUR: A50). An evidence-based decision tool is now available to help researchers consider these options (ARCHE: A3).

#### Should an overview of reviews include non-Cochrane systematic reviews?

Few research groups provided guidance on whether overviews of reviews should be restricted to Cochrane systematic reviews (CHF, CMIMG, EPPI, JBI). Two groups associated with Cochrane advocate for including only Cochrane systematic reviews if possible, but non-Cochrane systematic reviews might be considered if the available Cochrane reviews do not cover all of the important interventions (CHF: A15, CMIMG: A27). Two other groups advocate for the inclusion of both Cochrane and non-Cochrane systematic reviews, to ensure the breadth of coverage that is desired in the overview (EPPI: A40, JBI: A44). Including non-Cochrane systematic reviews increases comprehensiveness, but these systematic reviews might be of lower quality with less detailed reporting, and are likely to introduce primary study overlap, which adds complexity to the overview of reviews (CHF: A15, CMIMG: A27).

#### Assessing the quality or risk of bias of the included systematic reviews

Much of the available guidance indicates that it is important to appraise the quality of the included systematic reviews using a validated tool (ARCHE, KCE, CHF, CMIMG, CSU, EPOC, CPHG, DukeU, EPPI, HarvU, JBI, KCL, NEST, NOKC, SUR, TCD, UBirm, UConn, WJNR, UCyp). Several groups recommend independent assessment by at least two reviewers, with a process for resolving discrepancies (CMIMG: A27, CPHG: A38, DukeU: A39, JBI: A44, NEST: A48, NOKC: A49). Two groups recommend pilot testing (CMIMG: A27, UConn: A53), and another notes that authors should develop pre-defined decision rules (ARCHE: A2). There was no consensus on the ideal tool to use; fourteen groups mentioned AMSTAR (ARCHE: A2, KCE: A4, CHF: A15, EPOC: A37, CPHG: A38, DukeU: A39, JBI: A44, KCL: A47, NEST: A48, SUR: A50, TCD: A51, UConn: A53, WJNR: A57, UCyp: A55), with more recent guidance emphasizing AMSTAR 2 and ROBIS (KCE: A4, CHF: A13, JBI: A44, UConn: A53) which were released in 2017 and 2016 respectively. One group recommends assessing the quality of the systematic reviews as a whole, awarding points only if the amount and quality of information is sufficient for use at the overview of reviews level, and in the case of systematic reviews with multiple research questions, assessing only the quality for the comparison-outcome of interest for the overview of reviews (ARCHE: A2).

#### Collecting and presenting data on descriptive characteristics of included systematic reviews (and their primary studies)

Several groups recommend that data on descriptive characteristics be collected independently by at least two reviewers, with a process in place for resolving discrepancies (CMIMG: A27, EPOC: A37, JBI: A44, SUR: A50, UConn: A53). One group indicates that one reviewer with verification might occasionally be adequate (SUR: A50), and five recommend using a pilot-tested form (CMIMG: A27, EPOC: A37, JBI: A44, SUR: A50, UConn: A53, WJNR: A57). Important data to be collected from the systematic reviews includes citation details, search information, objectives, populations, setting, scope, risk of bias tool used, analysis methods, and outcomes of the included systematic reviews, as well as information about their included studies (KCE: A4, CHF: A15, CMIMG: A27, JBI: A44, NEST: A48, SUR: A50, TCD: A51). Descriptive characteristics of the systematic reviews should be presented narratively and/or in a table in adequate detail to support each systematic review’s inclusion in the overview of reviews, and inform the applicability of their findings (CHF: A15, CMIMG: A27, EPOC: A37, JBI: A44, NEST: A48, SUR: A50).

#### Collecting and presenting data on quality of primary studies contained within the included systematic reviews

Available guidance documents specify the importance of collecting and presenting data on the quality of the primary studies contained within the included systematic reviews (KCE, CHF, CMIMG, DukeU, EPPI, HarvU, JBI, NOKC, SUR, WJNR), but specific direction on how to do so is scant and conflicting. Six groups recommend preferentially extracting risk of bias assessments directly as reported in the included systematic reviews (CMIMG: A27, CHF: A15, NOKC: A49, EPPI: A40, SUR: A50, WJNR: A57). Three groups provide advice on dealing with systematic reviews that fail to report quality assessments, or assessments that seem unreliable, are discordant, or have been done using heterogeneous tools (CHF: A15, CMIMG: A27, SUR: A50). In these cases, authors could consider supplementing existing quality assessments (i.e., performing assessments for studies where this information is missing), or re-doing all quality assessments at the overview of reviews level (CHF: A15, CMIMG: A27, SUR: A50). One group indicates that it is important to extract and present domain-specific assessments when possible (CMIMG: A27), while others indicate that a summary of overall quality would be adequate (JBI: A44, NOKC: A49, SUR: A50).

#### Collecting, analyzing, and presenting outcome data

Several groups recommend that data be collected independently by at least two reviewers, with a process in place for resolving discrepancies (CMIMG: A27, EPOC: 37, JBI: A44, SUR: A50, UConn: A53). One group indicates that one reviewer with verification might occasionally be adequate (SUR: A50), and five recommend using a pilot-tested form (CMIMG: A27, EPOC: A37, JBI: A44, SUR: A50, UConn: A53, WJNR: A57). Most guidance documents recommend extracting data from the systematic reviews themselves (KCE: A4, CHF: A15, CMIMG: A27, EPOC: A37, EPPI: A40, JBI: A44, SUR: A50, UConn: A53). However, it is also noted that when important information is missing, authors may consider contacting systematic review authors, re-extracting data directly from the primary studies, or simply acknowledging the missing information (KCE: A4, CHF: A15, CMIMG: A27, EPPI: A40, JBI: A44, SUR: A50, UConn: A53). Prior to embarking on synthesis, twelve groups highlight the importance of authors investigating systematic reviews for primary study overlap, to avoid double-counting (KCE: A4, CHF: A15, CMIMG: A27, EPOC: A37, DukeU: A39, EPPI: A40, JBI: A44, SUR: A50, TCD: A51, UConn: A54, WHU: A58). Four groups recommend developing a citation matrix to visually map overlap, and calculating the corrected covered area (CCA) (CChile: A17, CMIMG: A27, UConn: A54, WHU: A58). Recent guidance recommends further investigation by calculating the CCA per pair of systematic reviews (CChile: A17) or per outcome (CMIMG: A27, UConn: A54), and examining overlapping systematic reviews further to understand whether reasons for overlap and/or discordant findings can be established (UConn: A54). An explanation about the size and number of overlapping studies, and the weight that these contribute to each analysis should be included in the presentation of results and/or discussion (CChile: A17, CMIMG: A27, WHU: A59).

The available guidance recommended two main methods of data analysis and presentation. The first is to simply summarize the data as they are originally presented within the systematic reviews (KCE: A4, CSU: A5, CHF: A15, CMIMG: A27, EPOC: A37, DukeU: A39, EPPI: A40, HarvU: A43, JBI: A44, NEST: A48, NOKC: A49, SUR: A50, WJNR: A57). If choosing this approach, it can be helpful to convert the results presented across the systematic reviews to one common summary statistic (CSU: A5, CMIMG: A27, HarvU: A43, SUR: A50, UConn: A53, UCyp: 55). The second method is to re-analyze the data in a different way than it has been analyzed and presented in the included systematic reviews (CHF: A15, CMIMG: A27, EPOC: A37, DukeU: A39, EPPI: A40, HarvU: A43, KCL: A47, SUR: A50, TCD: A51, UBirm: A52, UCyp: A55). Guidance from Cochrane recommends presenting the outcome data in a way that prevents making informal indirect comparisons across the systematic reviews (CMIMG: A27). One guidance document recommended that a brief, easily accessible, and easy to share summary of the evidence should be made available (GCU: A42).

#### Assessing the certainty/quality of the body of evidence

Most (*n* = 10/13, 77%) of the available guidance recommends using the Grading of Recommendations Assessment, Development, and Evaluation (GRADE) approach to appraise the certainty of the body of evidence (KCE: A4, CHF: A15, CMIMG: A27, DukeU: A39, JBI: A44, NEST: A48, NOKC: A49, SUR: A50, TCD: A51, WHU: A59), though formal guidance on how to apply GRADE in the context of an overview of reviews is not yet available (SUR: A50, WHU: 59). Two groups indicate that GRADE appraisals should be presented for each pre-defined important outcome (CMIMG: A27, CHF: A15). Three groups indicate that GRADE assessments should ideally be extracted directly from the included systematic reviews, but when these are unavailable, authors might consider re-doing GRADE assessments themselves (CHF: A15, CMIMG: A27, SUR: A50). One group (CMIMG: A27) indicated that authors might also need to consider re-performing GRADE appraisals when data have been re-analyzed, the scope of the overview of reviews differs from the included systematic reviews, or there are concerns about the quality of the appraisals presented (CMIMG: A27).

#### Interpreting outcome data and drawing conclusions

Guidance on interpreting outcome data and drawing conclusions is relatively sparse. The available documents indicate that conclusions should provide direct answers to the overview of review’s objectives (JBI: A44), comment on the quality and quantity of available information (KCE: A4, EPOC: A37, DukeU: A39, HarvU: A43, WJNR: A57), and be warranted based on the strengths and weaknesses of the included systematic reviews and their findings (KCE: A4, EPOC: A37, DukeU: A39, JBI: A44, WJNR: A57). Recommendations for both research and practice should be provided (JBI: A44, WJNR: A57). As previously mentioned in the “Collecting, analyzing, and presenting outcome data” section, authors should not make informal indirect comparisons across systematic reviews, or use wording that may encourage readers to make these types of comparisons (CMIMG: A27). Authors should indicate whether further research is likely to alter the conclusions (WHU: A59), or whether no further research is needed (WJNR: A57, WHU: A59).

#### Updating the overview

There are few guidance documents that address updating the overview; the ones that exist indicate that overviews of reviews should be regularly updated (CMIMG, GCU, SUR), but how and when this should be done is unclear. One group recommends that overviews of reviews should be updated when the conclusions of any of their included systematic reviews change, or new systematic reviews of relevance are published (CMIMG: A27). It is unclear how authors would keep apprised of such occurrences.

### Challenges

Since the previous version of this review [9], we identified 9 new documents identifying challenges related to undertaking overviews of reviews of healthcare interventions (A17, A47, A60, A66, A70, A71, A74, A75, A76). The challenges described in these documents, in addition to those from methodological guidance documents, expand upon those previously reported in Pollock et al.’s 2016 review [12]. The majority of documents report on challenges related to selecting systematic reviews, and potentially primary studies, for inclusion (*n* = 15); collecting and presenting descriptive characteristics (*n* = 11); assessing the certainty of evidence (*n* = 11); and collecting, analyzing, and presenting outcome data (*n* = 23). These challenges tend to mirror areas in which consensus remains lacking among currently available guidance. In particular, authors are still challenged by whether to include primary studies in their overviews, and how best to identify, address, and present information about primary study overlap either at the study selection or data extraction and analysis phases of the overview of reviews. A summary of all reported challenges is shown in Table 4.

**Table 4:** Reported challenges related to conducting overviews

Topic	Number of groups reporting challenges	Summary of reported challenges
Challenges related to the context for conducting overviews (i.e., when and why should you conduct an overview?		
Choosing between conducting an overview and a systematic review	2 (CHF, CMIMG)	It is not clear how to decide when it is better to perform an intervention systematic review versus an overview of reviews. It can be difficult to compare multiple interventions in the overview format, and it is often not feasible or appropriate to conduct a network-meta-analysis within an overview of reviews.
What types of questions about healthcare interventions can be answered using the overview format?	2 (CCG, CMG)	Methodological approaches may differ depending on the type of question that the overview of reviews aims to answer.
Questions to consider before deciding to conduct an overview	8 (CCRG, CHF, CMIMG, EPOC, JBI, UBirm, UCalg, UDun)	Overviews of reviews can be time consuming to produce, so there is a need to think about time and resource limitations and the need to balance flexibility with rigor. Authors need to think about the coverage and up-to-dateness of the available systematic reviews and decide whether an overview of reviews should be conducted if key primary studies or important interventions are missing from the available systematic reviews. Authors need to think about whether it would be feasible within time and resource constraints to update any systematic reviews that are out of date.
Author team composition and roles	2 (CMIMG, UCalg)	Authors are challenged with determining the size, composition, and skillset of the team members. A larger team than originally thought might be needed when individual contributors are limited in time.
Target audience of the overview	1 (CCG)	Approaches to preparing the overview of reviews may need to be adapted depending on the intended audience.
Challenges related to the process of conducting overviews (i.e., how do you conduct an overview?)		
Specifying the scope	9 (CHF, DCC, EPPI, LBI, RRI, UCalg, UBirm, UDun, WJNR)	Defining the scope and selecting and prioritizing populations and outcomes of interest can be difficult. The scope of available systematic reviews may be broader or narrower than the scope of the overview of reviews, and the available systematic reviews might not present data that are most relevant to the objective of the overview of reviews. When the scope is broad but time is limited, important outcomes might need to be prioritized.
Searching for systematic reviews	7 (CHF, CPHG, DukeU, EPOC, LBI, UBirm, UCalg)	Developing searches and deciding which index terms to use, which sources to search, and what restrictions should be placed on the search (e.g., language, date) can be challenging and need to be well thought out to avoid missing important systematic reviews. There is debate about the need to also search for primary studies that are not contained in any included systematic reviews, or when searches should be updated to find new primary studies. This adds complexity to the search.
Selecting systematic reviews for inclusion	15 (ARCHE, CHF, CMIMG, CSG, CSU, DukeU, EPOC, EPPI, GCU, JBI, RRI, UCalg, UBirm, UDun, WHU)	There are many decision points in selecting systematic reviews for inclusion that can be challenging and time-consuming. Authors need to decide how to define a ‘systematic review’, for which there is no single agreed upon definition. Authors then need to plan how they will handle systematic reviews that are out of date. They can update these themselves, add relevant primary studies, or concede that the findings of recent trials will be omitted. This can be a trade-off between amount and quality of evidence included. Finally, authors are challenged with identifying and handling primary study overlap at the selection level when many overlapping systematic reviews may exist. This can be time-intensive and challenging because of variable reporting across the available systematic reviews (e.g., may not transparently report all associated publications, may include different arms of the same trials).
Should an overview include non-Cochrane systematic reviews	3 (ARCHE, CHF, CMIMG)	The decision about whether to only include Cochrane systematic reviews or to also include non-Cochrane systematic reviews can be a balance between ensuring quality and coverage of all important interventions. Though non-Cochrane reviews can be of poorer methodological quality and have less detailed reporting, Cochrane reviews alone may not cover all relevant interventions or be adequately up to date. If authors choose to include both Cochrane and non-Cochrane systematic reviews, it is likely that they will need to deal with primary study overlap. However, this may occur even if only Cochrane systematic reviews are included.
Assessing the quality of included systematic reviews	10 (ARCHE, CCRG, CHF, CMIMG, EPPI, EPOC, GCU, PXU, RRI, UDun)	There is no agreement on which tool might be best to use (e.g., AMSTAR, AMSTAR 2, or ROBIS) to assess methodological quality, or how to use them in the context of an overview of reviews. It can be difficult to distinguish between methodological quality and the quality of reporting, and poor reporting in the systematic reviews can make assessment challenging. Authors often have difficulty interpreting and coming to agreement with assessments on the available tools. It is unclear whether authors should assess systematic reviews in their entirety or only the components that are relevant to the overview question, and what to do with systematic reviews that include other embedded reviews. When overview quality is being used to choose between overlapping systematic reviews, authors need to be careful to not exclude potentially relevant information. When overlapping systematic reviews use different methodologies and come to discordant conclusions, it can be hard to tell whether their methods are appropriate.
Collecting and presenting data on descriptive characteristics of included systematic reviews (and primary studies)	11 (CCRG, CHF, CMIMG, CMG, DCC, DukeU, EPOC, JBI, LBI, NOKC, UCalg)	Overview authors are challenged with data extraction at two levels, first the level of the systematic review, and then potentially the level of the primary study. When relying on the reporting of the included systematic reviews, authors may struggle when these are poorly reported and missing important details. Overview authors need to carefully check systematic reviews for errors in data extraction, as these errors will lead to errors in the overview of reviews. They also need to decide how to deal with systematic reviews with missing information of relevance to the overview of reviews. Going back to the primary studies can be time consuming, but not doing so can lead to a loss of information.
Collecting and presenting data on quality of primary studies contained within included systematic reviews	7 (CCRG, CHF, CSG, DCC, EPOC, EPPI, JBI)	Collecting and presenting information on the quality of the primary studies can mean relying on the appraisals of the original systematic review authors, which may be flawed, inconsistent, or poorly reported. Some systematic reviews may only report a summary of appraisals, rather than the risk of bias or quality of individual studies or outcomes of interest. Comparisons across systematic reviews can be difficult if different tools are used in each, because using different methods of assessing risk of bias can lead to disparate judgments.
Collecting, analyzing, and presenting outcome data	23 (ARCHE, CChile, CCRG, CMG, CMIMG, CHF, DCC, DukeU, EPOC, EPPI, JBI, KCL, LBI, NOKC, PXU, RRI, TCD, UAuck, UBirm, UCalg, UDun, WHU, WJNR)	Many difficulties may arise when collecting, analyzing, and presenting findings at the overview level, because of inconsistency in methodology and reporting of findings across systematic reviews. For example, the included systematic reviews and their primary studies may use heterogeneous outcome measures. Additionally, the included systematic reviews may be incompletely reported, or may not report data on subgroups of interest. Overlapping systematic reviews might present discordant results or present similar data in different ways (e.g., different summary measures), and it can be complex and time-consuming to ensure that data from single studies are not over-represented. Interpretation of measures of overlap (e.g., matrices and corrected covered area) can be a challenge when the number of primary studies is large. To perform analyses of interest, overview authors might need to go back to individual studies, or concede that the available information is incomplete. It may not always be appropriate or feasible to conduct meta-analyses in overviews, and network meta-analyses and informal indirect comparisons are usually not appropriate. However, narrative synthesis can become complex and open to bias if not adequately described. There is a concern that synthesis errors at the SR level could result in errors at the overview level.
Assessing quality of evidence of outcome data	11 (CCRG, CHF, CMG, CMIMG, CSG, DCC, EPOC, Glasgow, PXU, RRI, UDun)	It may not be possible or appropriate to simply extract existing GRADE appraisals from the included systematic reviews. The reviews might not include GRADE appraisals for the outcomes or populations of interest or be missing details on each of the GRADE considerations. Different systematic reviews with the same studies that have made different decisions about handling data (analysis) and appraising study quality may come to different GRADE conclusions, especially related to the study limitations, consistency, and precision domains. Different raters across systematic reviews could come to different conclusions, due to the subjectivity of the GRADE approach. If re-doing the GRADE for each systematic review, authors are likely to encounter difficulty due to an absence of guidance on how to apply GRADE in the context of an overview, incomplete reporting at the level of the systematic review, and a lack of familiarity with the contributing primary studies.
Interpreting outcome data and drawing conclusions	9 (CHF, CMIMG, DCC, DukeU, EPOC, GCU, LBI, URRI, UCalg)	Interpreting data and drawing conclusions can be difficult. The included systematic reviews (and their included primary studies) may use heterogeneous outcome measures which can limit the ability to draw useful conclusions. Procedural variation at the systematic review and overview levels (e.g., study selection, data extraction) can lead to different conclusions from the same set of data. It can be difficult to provide interpretation of analyses of multiple interventions; multiple comparisons from different systematic reviews that are included in the same overview; discordant results and conclusions across the included systematic reviews. Authors need to consider the methods used in the systematic reviews and overview, and decide how best to highlight uncertainties and gaps that remain.

## Discussion

This scoping review has revealed a steady accumulation of new guidance and provides a single source where author teams can locate information to help them decide if, when, and how to embark on an overview of reviews. New guidance that has become available over the past 5 years has helped to resolve some common challenges inherent in the production of overviews of reviews. Important developments include a decision tool for selecting systematic reviews for inclusion in overviews of reviews [25] and expanded guidance on handling primary study overlap at the analysis stage [26, 27]. Despite marked progress, several areas continue to be characterized by inconsistent or insufficient guidance. For example, there is ongoing debate about whether, when, and how supplemental primary studies should be included in overviews of reviews. Empirical evidence is lacking on the optimal tool for assessing risk of bias or methodological quality of included systematic reviews, and how these tools might best be applied in overviews of reviews [28, 29]. Guidance remains limited on how to extract and use appraisals of the quality of primary studies within the included systematic reviews and how to adapt GRADE methodology to overviews of reviews [7, 21]. The challenges that overview authors reportedly face are often related to the steps where guidance is inadequate or conflicting.

Authors report facing challenges in the more complex steps of the overview process (and those that may differ most from systematic reviews), where guidance is either lacking (e.g., how to apply GRADE methodology to overviews) or where there is still no consensus on the preferred approach (e.g., how to best identify, manage, and present information on overlap). When guidance is available, it most often enumerates options on how to deal with these challenges that balance methodological rigor, comprehensiveness, and feasibility. There is insufficient empirical evidence, however, to fully understand how many of these methodological decisions may impact reviewer workload, the validity of results and conclusions of overviews of reviews, and their relevance for healthcare decision-makers [30]. Since there does not yet exist a minimum standard of conduct and reporting, published overviews of reviews use highly heterogeneous methodologies [11, 30–32] and are often poorly and inconsistently reported [4, 5, 10]. The propagation of substandard overviews of reviews has the potential to undermine their legitimacy as an important tool for healthcare decision-making, and substantiates the urgent need to develop evidence-based conduct and reporting standards akin to what exists for systematic reviews [33, 34]. Studies evaluating the impact of methodological decisions on the aforementioned outcomes have recently begun to emerge [35]. Authors would benefit from practical decision tools to guide them through the rigor-to-feasibility trade-offs that are common in overviews of reviews.

Researchers wishing to undertake an overview of reviews of healthcare interventions in 2020 are still challenged by a fragmented body of guidance documentation, but this should not overshadow the substantial developments in the science of overviews of reviews that have occurred over the past few years. In particular, both Cochrane [18] and the Joanna Briggs Institute [36] have released much needed updated handbook chapters that incorporate the most recent empirical evidence for producing overviews of reviews. Authors may use these stand-alone guidance documents to inform the planning of all stages of the overview of reviews. A decision tool published in 2019 can help researchers make informed decisions about managing primary study overlap at the selection stage of the overview of reviews [25]. How overview of reviews authors might best explore and present data on primary study overlap has become an area of increased research interest [26, 27, 37, 38]. An evidence-based and consensus-based reporting guideline for overviews of reviews is currently in development [9]. The ongoing synthesis of accruing guidance for overviews of reviews, and primary research studies assessing the impact of methodological decisions in the more highly debated steps of overviews of reviews, will support the development of an evidence-based and consensus-based set of minimum methodological expectations for their conduct. The development of these minimum standards will, in turn, help overview authors to overcome many of the current challenges in the overview process.

Producing overviews of reviews is inherently demanding given the need to make sense of multiple levels of evidence (i.e., the systematic review level and primary study level) and overcome challenges for which there is often no agreed-upon solution [7]. One of the proposed advantages of overviews of reviews is that they can create efficiencies by making use of evidence already compiled in systematic reviews [6, 7]. As guidance has accrued to assist authors in surmounting common challenges, however, it has become increasingly clear that suggested methods for undertaking overviews of reviews can require substantial expertise, time, and resources. Indeed, authors report challenges at all phases of the overview process. Data extraction, quality appraisal, and synthesis of data from systematic reviews can be extremely challenging and time-consuming because the reporting quality of systematic reviews is highly variable [2]. When authors are unable to extract all of the desired information from systematic reviews themselves, they may decide to return to the primary studies, but this can extend the timeline and overall work required for the overview substantially. Otherwise, authors must accept that the overview may be missing important information. Even when extracting information directly from the available systematic reviews, making sense of discordant results and conclusions can be tedious. For these reasons, it is important for authors to develop a good understanding of the available systematic reviews before embarking on the overview, and plans to deal with missing or discordant information should be devised at the protocol stage [2, 32–34].

### Strengths and limitations

We used a transparent and rigorous approach to summarize information from all available guidance documents for overviews of reviews of healthcare interventions, and reports of author experiences. The guidance summarized herein may not be directly applicable to other types of overviews of reviews (e.g., diagnostic accuracy, qualitative). We used the search strategies that offered the highest yield in the original version of this scoping review, and located much of the guidance within the grey literature (e.g., websites, conference proceedings). It is possible that some guidance has been missed by not employing term-based databased searches, and that the results may have differed if another set of seed articles were used. We limited this possibility by employing an iterative and rigorous search strategy (i.e., alerts in multiple databases and hand-searching multiple sources).

## Conclusion

The rising popularity of overviews of reviews has been accompanied by a steady accumulation of new and sometimes conflicting guidance, yet several areas of uncertainty remain. These findings are being used to inform the development of a reporting guideline for overviews of reviews, which aims to support the high quality and transparency of reporting that is needed to substantiate overviews as a robust source of evidence for healthcare decision-making. Empirical research is needed to provide the data necessary to support the development of a minimum set of methodological expectations for the conduct of overviews of reviews.

## Supplementary Information

**Additional file 1.** PRISMA-ScR checklist. Completed reporting checklist for scoping reviews.

**Additional file 2.** Details of the search strategies.

**Additional file 3.** Studies excluded following full text review.

**Additional file 4.** Documents included in the scoping review.

## Data Availability

The datasets analyzed during the current study are available from the corresponding author on reasonable request.

## Abbreviations

CCA: Corrected covered area
GRADE: Grading of Recommendations, Assessment, Development and Evaluation
PRIOR: Preferred Reporting Items for Overviews of Reviews
