# Supplementary material for: Guidance for overviews of reviews continues to accumulate, but important challenges remain: a scoping review
Source: Syst Rev. 2020 Nov 4;9:254. doi: 10.1186/s13643-020-01509-0 (PMC7643411; doi:10.1186/s13643-020-01509-0)
Supplement: Supplementary file 2 — Additional file 2. Details of the search strategies. [file 13643_2020_1509_MOESM2_ESM.docx]

**ELECTRONIC SEARCH STRATEGIES**

1. **Reference Tracking**

**Start Date:** 7-8 March 2019 (with retrospective searches 2014-present)

**End Date:** 31 March 2020

**Procedure*:** For each target article, we searched for "citing" references (Google Scholar, Scopus), "cited" references (reference lists), and "similar articles" (PubMed).

**Target Articles (n = 46):**

Aromataris E, Fernandez R, Godfrey CM, Holly C, Khalil H, Tungpunkom P. Summarizing systematic reviews: methodological development, conduct and reporting of an umbrella review approach. International Journal of Evidence-Based Healthcare. 2015;13(3):132-40. PMID: 26360830

Aromataris E, Fernandez RS, Godfrey C, Holly C, Khalil H, Tungpunkom P. Methodology for JBI umbrella reviews. In: The Joanna Briggs Institute Reviewers Manual 2014. Adelaide, Australia: The Joanna Briggs Institute; 2014.

Baker PRA, Costello JT, Dobbins M, B. Waters E. The benefits and challenges of conducting an overview of systematic reviews in public health: a focus on physical activity. Journal of Public Health. 2014;36(3):517-21. PMID: 25085438

Ballard M, Montgomery P. Risk of bias in overviews of reviews: A scoping review of methodological guidance and four-item checklist. Research Synthesis Methods. 2017;8(1):92-108. PMID: 28074553

Becker LA, Oxman AD. Chapter 22: Overviews of reviews. In: Higgins JPT, Green S (editors). Cochrane handbook for systematic reviews of interventions (version 5.1.0). London, UK: The Cochrane Collaboration; 2011.

Bougioukas KI, Bouras E, Apostolidou-Kiouti F, Kokkali S, Arvanitidou M, Haidich AB. Reporting guidelines on how to write a complete and transparent abstract for overviews of systematic reviews of health care interventions. Journal of Clinical Epidemiology. 2019;106:70-9. PMID: 30336211

Bougioukas KI, Liakos A, Tsapas A, Ntzani E, Haidich AB. Preferred reporting items for overviews of systematic reviews including harms checklist: A pilot tool to be used for balanced reporting of benefits and harms. Journal of clinical epidemiology. 2018;93:9-24. PIMD: 29037888

Buchter RB, Pieper D. Most overviews of Cochrane reviews neglected potential biases from dual authorship. Journal of Clinical Epidemiology. 2016;77:91-4. PMID: 27131430

Caird J, Sutcliffe K, Kwan I, Dickson K, Thomas J. Mediating policy-relevant evidence at speed: Are systematic reviews of systematic reviews a useful approach? Evidence & Policy. 2015;11(1):81-97.

Caldwell DM, Welton NJ, Ades AE. Mixed treatment comparison analysis provides internally coherent treatment effect estimates based on overviews of reviews and can reveal inconsistency. Journal of Clinical Epidemiology. 2010;63(8):875-82. PMID: 20080027

Conn VS, Coon Sells TG. WJNR welcomes umbrella reviews. Los Angeles, CA: Sage Publications; 2014. PMID: 24391147

Cooper H, Koenka AC. The overview of reviews: unique challenges and opportunities when research syntheses are the principal elements of new integrative scholarship. The American Psychologist. 2012;67(6):446-62. PMID: 22352742

Crick K, Wingert A, Williams K, Fernandes RM, Thomson D, Hartling L. An evaluation of harvest plots to display results of meta-analyses in overviews of reviews: A cross-sectional study. BMC Medical Research Methodology. 2015;15(1):91. PMID: 26502717

Elliott L, Crombie IK, Irvine L, Cantrell J, Taylor J. The effectiveness of public health nursing: the problems and solutions in carrying out a review of systematic reviews. Journal of Advanced Nursing. 2004;45(2):117-25. PMID: 14705995

Esposito M. Editorial: Overviews and umbrella reviews. European Journal of Oral Implantology. 2018;11(3):255. PIMD: 30246180

Faggion CM, Jr., Cavero KD. Overview authors rarely defined systematic reviews that are included in their overviews. Journal of Clinical Epidemiology. 2019. S0895-4356(18)30640-1. PMID: 30684566

Fusar-Poli P, Radua J. Ten simple rules for conducting umbrella reviews. Evidence-based Mental Health. 2018;21(3):95-100. PMID: 30006442

Hartling L, Chisholm A, Thomson D, Dryden DM. A descriptive analysis of overviews of reviews published between 2000 and 2011. PloS One. 2012;7(11):e49667. PMID: 23166744

Hartling L, Vandermeer B, Fernandes RM. Systematic reviews, overviews of reviews and comparative effectiveness reviews: A discussion of approaches to knowledge synthesis. Evidence-based Child Health: a Cochrane Review Journal. 2014;9(2):486-94. PMID: 25404611

Hemming K, Bowater RJ, Lilford RJ. Pooling systematic reviews of systematic reviews: A Bayesian panoramic meta‐analysis. Statistics in Medicine. 2012;31(3):201-16. PMID: 21965138

Hunt H, Pollock A, Campbell P, Estcourt L, Brunton G. An introduction to overviews of reviews: planning a relevant research question and objective for an overview. Systematic Reviews. 2018;7(1):39. PMID: 29490699

Ioannidis J. Next-generation systematic reviews: prospective meta-analysis, individual-level data, networks and umbrella reviews. British Journal of Sports Medicine. 2017;51(20):1456-8. PMID: 28223307

Li L, Tian J, Tian H, Sun R, Liu Y, Yang K. Quality and transparency of overviews of systematic reviews. Journal of Evidence-based Medicine. 2012;5(3):166-73. PMID: 23672223

Lunny C, Brennan SE, McDonald S, McKenzie JE. Toward a comprehensive evidence map of overview of systematic review methods: Paper 1-purpose, eligibility, search and data extraction. Systematic Reviews. 2017;6(1):231. PMID: 29162130

Lunny C, Brennan SE, McDonald S, McKenzie JE. Toward a comprehensive evidence map of overview of systematic review methods: Paper 2-risk of bias assessment; synthesis, presentation and summary of the findings; and assessment of the certainty of the evidence. Systematic Reviews. 2018;7(1):159. PMID: 30314530

McKenzie JE, Brennan SE. Overviews of systematic reviews: great promise, greater challenge. Systematic Reviews. 2017;6(1):185. PMID: 28886726

Pieper D, Antoine SL, Morfeld JC, Mathes T, Eikermann M. Methodological approaches in conducting overviews: Current state in HTA agencies. Research Synthesis Methods. 2014;5(3):187-99. PMID: 26052845

Pieper D, Antoine S-L, Mathes T, Neugebauer EA, Eikermann M. Systematic review finds overlapping reviews were not mentioned in every other overview. Journal of Clinical Epidemiology. 2014;67(4):368-75. PMID: 24581293

Pieper D, Antoine S-L, Neugebauer EA, Eikermann M. Up-to-dateness of reviews is often neglected in overviews: A systematic review. Journal of Clinical Epidemiology. 2014;67(12):1302-8. PMID: 25281222

Pieper D, Buchter RB, Antoine SL, Eikermann M. [Overviews - status quo, potentials and perspectives]. Zeitschrift fur Evidenz, Fortbildung und Qualitat im Gesundheitswesen. 2013;107(9-10):592-6. PMID: 24315329

Pieper D, Buechter R, Jerinic P, Eikermann M. Overviews of reviews often have limited rigor: A systematic review. Journal of Clinical Epidemiology. 2012;65(12):1267-73. PMID: 22959594

Pieper D, Pollock M, Fernandes RM, Buchter RB, Hartling L. Epidemiology and reporting characteristics of overviews of reviews of healthcare interventions published 2012-2016: Protocol for a systematic review. Systematic Reviews. 2017;6(1):73. PMID: 28388960

Pieper D, Waltering A, Holstiege J, Buchter RB. Quality ratings of reviews in overviews: A comparison of reviews with and without dual (co-)authorship. Systematic Reviews. 2018;7(1):63. PMID: 29690911

Piso B, Semlitsch T, Reinsperger I, Breuer J, Kaminski-Hartenthaler A, Kien C, et al. [Practical experience with overviews of reviews--valuable decision aid or academic exercise?]. Zeitschrift fur Evidenz, Fortbildung und Qualitat im Gesundheitswesen. 2015;109(4-5):300-8. PMID: 26354130

Pollock A, Campbell P, Brunton G, Hunt H, Estcourt L. Selecting and implementing overview methods: implications from five exemplar overviews. Systematic Reviews. 2017;6(1):145. PMID: 28720141

Pollock M, Fernandes RM, Becker LA, Featherstone R, Hartling L. What guidance is available for researchers conducting overviews of reviews of healthcare interventions? A scoping review and qualitative metasummary. Systematic Reviews. 2016;5(1):190. PMID: 27842604

Pollock M, Fernandes RM, Hartling L. Evaluation of AMSTAR to assess the methodological quality of systematic reviews in overviews of reviews of healthcare interventions. BMC Medical Research Methodology. 2017;17(1):48. PMID: 28335734

Pollock M, Fernandes RM, Newton AS, Scott SD, Hartling L. The impact of different inclusion decisions on the comprehensiveness and complexity of overviews of reviews of healthcare interventions. Systematic Reviews. 2019;8(1):18. PMID: 30635048

Pollock M, Fernandes RM, Newton AS, Scott SD, Hartling L. A decision tool to help researchers make decisions about including systematic reviews in overviews of reviews of healthcare interventions. Systematic Reviews. 2019;8(1):29. PMID: 30670086

Ryan RE, Kaufman CA, Hill SJ. Building blocks for meta-synthesis: data integration tables for summarising, mapping, and synthesising evidence on interventions for communicating with health consumers. BMC Medical Research Methodology. 2009;9(1):16. PMID: 19261177

Schultz A, Goertzen L, Rothney J, Wener P, Enns J, Halas G, et al. A scoping approach to systematically review published reviews: Adaptations and recommendations. Research Synthesis Methods. 2018;9(1):116-23. PMID: 29032590

Silva V, Grande AJ, Carvalho AP, Martimbianco AL, Riera R. Overview of systematic reviews - a new type of study. Part II. Sao Paulo Medical Journal. 2015;133(3):206-17. PMID: 25388685

Silva V, Grande AJ, Martimbianco AL, Riera R, Carvalho AP. Overview of systematic reviews - a new type of study: part I: why and for whom? Sao Paulo Medical Journal. 2012;130(6):398-404. PMID: 23338737

Smith V, Devane D, Begley CM, Clarke M. Methodology in conducting a systematic review of systematic reviews of healthcare interventions. BMC Medical Research Methodology. 2011;11(1):15. PMID: 21291558

Thomson D, Foisy M, Oleszczuk M, Wingert A, Chisholm A, Hartling L. Overview of reviews in child health: evidence synthesis and the knowledge base for a specific population. Evidence‐Based Child Health: A Cochrane Review Journal. 2013;8(1):3-10. PMID: 23878121

Thomson D, Russell K, Becker L, Klassen T, Hartling L. The evolution of a new publication type: Steps and challenges of producing overviews of reviews. Research Synthesis Methods. 2010;1(3‐4):198-211. PMID: 26061466

* Procedure modified from: Horsley T, Dingwall O, Sampson M. Checking reference lists to find additional studies for systematic reviews. Cochrane Database Syst Rev. 2011;(8):MR000026, and Greenhalgh T, Peacock R. Effectiveness and efficiency of search methods in systematic reviews of complex evidence: audit of primary sources. BMJ. 2005;331(7524):1064-5.

**Search Details**

1. **PubMed (NCBI) similar articles**

On 7 March 2019 we ran a Similar Articles search for the target articles shown below (resulted in 2465 records) and restricted the date as shown below. The search was then run monthly on the first day of the month to locate new articles indexed in the previous month.

| 26360830[uid] or 25085438[uid] or 28074553[uid] or 30336211[uid] or 29037888[uid] or 27131430[uid] or 20080027[uid] or 30684566[uid] or 24391147[uid] or 22352742[uid] or 26502717[uid] or 14705995[uid] or 30246180[uid] or 30006442[uid] or 23166744[uid] or 25404611[uid] or 21965138[uid] or 29490699[uid] or 28223307[uid] or 23672223[uid] or 29162130[uid] or 30314530[uid] or 28886726[uid] or 26052845[uid] or 24581293[uid] or 25281222[uid] or 24315329[uid] or 22959594[uid] or 28388960[uid] or 29690911[uid] or 26354130[uid] or 28720141[uid] or 27842604[uid] or 28335734[uid] or 30635048[uid] or 30670086[uid] or 19261177[uid] or 29032590[uid] or 25388685[uid] or 23338737[uid] or 21291558[uid] or 23878121[uid] or 26061466[uid] |
| --- |

**The following target articles were not indexed in PubMed, and were excluded from the search:**

Aromataris E, Fernandez RS, Godfrey C, Holly C, Khalil H, Tungpunkom P. Methodology for JBI umbrella reviews. In: The Joanna Briggs Institute Reviewers Manual 2014. Adelaide, Australia: The Joanna Briggs Institute; 2014.

Becker LA, Oxman AD. Chapter 22: Overviews of reviews. In: Higgins JPT, Green S (editors). Cochrane handbook for systematic reviews of interventions (version 5.1.0). London, UK: The Cochrane Collaboration; 2011.

Caird J, Sutcliffe K, Kwan I, Dickson K, Thomas J. Mediating policy-relevant evidence at speed: Are systematic reviews of systematic reviews a useful approach? Evidence & Policy. 2015;11(1):81-97.

**Details of the PubMed Similar Articles Searches**

| **Date of search** | **Date limit applied** |
| --- | --- |
| 7 March 2019 | “2014/01/01”[CRDT]:”2019/03/07”[CRDT] |
| 1 April 2019 | “2019/03/07”[CRDT]:”2019/03/31”[CRDT] |
| 1 May 2019 | “2019/04/01”[CRDT]:”2019/04/30”[CRDT] |
| 1 June 2019 | “2019/05/01”[CRDT]:”2019/05/31”[CRDT] |
| 1 July 2019 | “2019/06/01”[CRDT]:”2019/06/30”[CRDT] |
| 1 August 2019 | “2019/07/01”[CRDT]:”2019/07/31”[CRDT] |
| 1 Sept 2019 | “2019/08/01”[CRDT]:”2019/08/31”[CRDT] |
| 1 Oct 2019 | “2019/09/01”[CRDT]:”2019/09/30”[CRDT] |
| 1 Nov 2019 | “2019/10/01”[CRDT]:”2019/10/31”[CRDT] |
| 1 Dec 2019 | “2019/11/01”[CRDT]:”2019/11/30”[CRDT] |
| 1 Jan 2020 | “2019/12/01”[CRDT]:”2019/12/31”[CRDT] |
| 1 Feb 2020 | “2020/01/01”[CRDT]:”2020/01/31”[CRDT] |
| 1 March 2020 | “2020/02/01”[CRDT]:”2020/02/29”[CRDT] |
| 1 April 2020 | “2020/03/01”[CRDT]:”2020/03/31”[CRDT] |

1. **Scopus Citing References**

On 8 March 2019 we ran a Cited References search for each target article and restricted the date to from 2014 to present. Then an e-mail alert was set for the first day of each month for each article as “PRIOR – Author Year”.

**The following target articles were not indexed in Scopus, and were excluded from the search:**

Aromataris E, Fernandez RS, Godfrey C, Holly C, Khalil H, Tungpunkom P. Methodology for JBI umbrella reviews. In: The Joanna Briggs Institute Reviewers Manual 2014. Adelaide, Australia: The Joanna Briggs Institute; 2014.

Becker LA, Oxman AD. Chapter 22: Overviews of reviews. In: Higgins JPT, Green S (editors). Cochrane handbook for systematic reviews of interventions (version 5.1.0). London, UK: The Cochrane Collaboration; 2011.

Thomson D, Russell K, Becker L, Klassen T, Hartling L. The evolution of a new publication type: Steps and challenges of producing overviews of reviews. Research Synthesis Methods. 2010;1(3‐4):198-211. PMID: 26061466

1. **Google Scholar Citing References**

On 8 March 2019 we searched each target article in Google Scholar and restricted the date as shown below.

**Details of the Google Scholar Citing References Searches**

| **Target article** | **Date searched** | **# cited references within database (with duplicates)** |
| --- | --- | --- |
| 1. Aromataris 2015 | 8 March 2019 | 131 |
| 2. Aromataris 2014 | 8 March 2019 | 27 |
| 3. Baker 2014 | 8 March 2019 | 21 |
| 4. Ballard 2014 | 8 March 2019 | 35 |
| 5. Becker 2011 | 8 March 2019 | 273 |
| 6. Bougioukas 2019a | 8 March 2019 | 0 |
| 7. Bougioukas 2019b | 8 March 2019 | 10 |
| 8. Buchter 2016 | 8 March 2019 | 9 |
| 9. Caird 2015 | 8 March 2019 | 37 |
| 10. Caldwell 2010 | 8 March 2019 | 109 |
| 11. Conn 2014 | 8 March 2019 | 5 |
| 12. Cooper 2012 | 8 March 2019 | 88 |
| 13. Crick 2015 | 8 March 2019 | 10 |
| 14. Elliott 2004 | 8 March 2019 | 12 |
| 15. Esposito 2018 | 8 March 2019 | 0 |
| 16. Faggion 2019 | 8 March 2019 | 0 |
| 17. Fusar-Poli 2018 | 8 March 2019 | 4 |
| 18. Hartling 2012 | 8 March 2019 | 91 |
| 19. Hartling 2014 | 8 March 2019 | 36 |
| 20. Hemming 2012 | 8 March 2019 | 12 |
| 21. Hunt 2018 | 8 March 2019 | 5 |
| 22. Ioannidis 2017 | 8 March 2019 | 21 |
| 23. Li 2012 | 8 March 2019 | 30 |
| 24. Lunny 2017 | 8 March 2019 | 7 |
| 25. Lunny 2018 | 8 March 2019 | 1 |
| 26. McKenzie 2017 | 8 March 2019 | 15 |
| 27. Pieper 2014a | 8 March 2019 | 15 |
| 28. Pieper 2014b | 8 March 2019 | 62 |
| 29. Pieper 2014c | 8 March 2019 | 20 |
| 30. Pieper 2013 | 8 March 2019 | 4 |
| 31. Pieper 2012 | 8 March 2019 | 83 |
| 32. Pieper 2017 | 8 March 2019 | 4 |
| 33. Pieper 2018 | 8 March 2019 | 0 |
| 34. Piso 2015 | 8 March 2019 | 3 |
| 35. Pollock A 2017 | 8 March 2019 | 16 |
| 36. Pollock 2016 | 8 March 2019 | 32 |
| 37. Pollock M 2017 | 8 March 2019 | 27 |
| 38. Pollock 2019a | 8 March 2019 | 1 |
| 39. Pollock 2019b | 8 March 2019 | 1 |
| 40. Ryan 2009 | 8 March 2019 | 36 |
| 41. Schultz 2018 | 8 March 2019 | 2 |
| 42. Silva 2015 | 8 March 2019 | 15 |
| 43. Silva 2012 | 8 March 2019 | 25 |
| 44. Smith 2011 | 8 March 2019 | 401 |
| 45. Thomson 2013 | 8 March 2019 | 18 |
| 46. Thomson 2010 | 8 March 2019 | 69 |
| **TOTAL** |  | **1823*** |

*1102 without duplicates.

1. **Google Scholar Search**

**Search Date:** 1 March 2019 (search strategy was then turned into an article alert; alerts were monitored as part of the update search up to 31 March 2020)

| (”review of reviews”\|”overview of systematic reviews”\|”review of systematic reviews”\|”systematic review of reviews”\|”overview of reviews”\|”umbrella review”\|”systematic overview”) (200)* |
| --- |

* The first 20 pages of results were reviewed. Limited to articles since 2014.

**HAND SEARCHES**

1. **Relevant websites**

**Search Date:** 7-12 February 2019

**Search updated:** 3-5 February 2020

**Websites Searched:** 59; Includes organizations known to have conducted at least one overview, and major evidence synthesis centres.

| **Website** | **URL** |
| --- | --- |
| Agency for Healthcare Research and Quality | https://www.ahrq.gov/ |
| Alberta Innovates | https://albertainnovates.ca |
| American Association of Diabetes Educators | https://www.diabeteseducator.org/ |
| British Dietetic Association | https://www.bda.uk.com/ |
| Cambridge Institute of Public Health | https://www.iph.cam.ac.uk/ |
| Campbell Collaboration | https://campbellcollaboration.org/ |
| Canadian Agency for Drugs and Technologies in Health | <http://www.cadth.ca/> |
| Canadian Institutes of Health Research | <http://www.cihr-irsc.gc.ca/e/193.html> |
| Canadian Task Force on Preventive Health Care | https://canadiantaskforce.ca |
| Centre for Evidence-based Health Care | https://www.nottingham.ac.uk/research/groups/cebhc/index.aspx |
| Centre for Evidence-based Medicine | https://www.cebm.net/ |
| Centre on Knowledge Translation for Disability and Rehabilitation Research | <https://ktdrr.org/training/webcasts/index.html> |
| Centre for Reviews and Dissemination | https://www.york.ac.uk/crd/ |
| Centro Cochrane do Brasil | https://brazil.cochrane.org/ |
| Cochrane | https://www.cochrane.org/ |
| Cochrane Acute Respiratory Infections Group | https://ari.cochrane.org/ |
| Cochrane Airways Group | https://airways.cochrane.org/ |
| 1. Cochrane Anaesthesia Group | https://ace.cochrane.org/ |
| Cochrane Child Health Field | <http://childhealth.cochrane.org/> |
| Cochrane Comparing Multiple Interventions Methods Group | <http://cmim.cochrane.org/> |
| Cochrane Consumers and Communications Group | <http://cccrg.cochrane.org/> |
| 1. Cochrane Cystic Fibrosis and Genetic Disorders Group | https://cfgd.cochrane.org/ |
| 1. Cochrane Developmental, Psychosocial and Learning Problems Group | https://dplp.cochrane.org/ |
| Cochrane Effective Practice and Organization of Care | <http://epoc.cochrane.org/> |
| 1. Cochrane Gynaecology and Fertility Group | https://cgf.cochrane.org/ |
| Cochrane Hepato-Biliary Group | <http://hbg.cochrane.org/> |
| Cochrane Incontinence Group | <http://incontinence.cochrane.org/> |
| Cochrane Multiple Sclerosis and Rare Diseases of the CNS Group | https://msrdcns.cochrane.org/ |
| Cochrane Musculoskeletal Group | <http://musculoskeletal.cochrane.org/> |
| 1. Cochrane Neonatal Group | https://neonatal.cochrane.org/ |
| Cochrane Neuromuscular Group | https://neuromuscular.cochrane.org/ |
| Cochrane Pain, Palliative and Supportive Care Group | https://papas.cochrane.org/ |
| 1. Cochrane Pregnancy and Childbirth Group | https://pregnancy.cochrane.org/ |
| Cochrane Schizophrenia Group | <http://szg.cochrane.org/> |
| 1. Cochrane Stroke Group | https://stroke.cochrane.org/ |
| 1. Cochrane Wounds Group | https://wounds.cochrane.org/ |
| Consiglio Nazionale delle Ricerche (Italy) | https://www.cnr.it/ |
| Department of Violence and Injury Prevention and Disability, World Health Organization | <http://www.who.int/violence_injury_prevention/en/> |
| EPPI centre | https://eppi.ioe.ac.uk/cms/Default.aspx?tabid=53 |
| Federal Institute for Occupational Safety and Health (BAuA), Berlin, Germany | https://www.baua.de/EN/Home/Home_node.html |
| Finnish Office for Health Technology Assessment and National Research and Development Centre for Welfare and Health | http://www.inahta.org/ |
| Health Protection Scotland | <http://www.hps.scot.nhs.uk/> |
| Iberoamerican Cochrane Group | <http://es.cochrane.org/es> |
| Institute for Clinical Effectiveness and Health Policy, Argentina | <http://www.iecs.org.ar/index.php> |
| International Association of Cancer Registries | http://www.iacr.com.fr/index.php |
| Independent Institute for Quality and Efficiency in Health Care (IQWIG) | <https://www.iqwig.de/en/home.2724.html> |
| Joanna Briggs Institute | <http://joannabriggs.org/> |
| McMaster University Health Systems Evidence | <http://www.healthsystemsevidence.org> |
| National Institute for Health Care Excellence (NICE) | https://www.nice.org.uk |
| Netherlands Institute for Health Services Research | <http://www.nivel.nl/en> |
| Netherlands Institute of Mental Health and Addiction | <http://www.trimbos.org/> |
| Norwegian Knowledge Centre for the Health Services | <http://www.kunnskapssenteret.no/home> |
| Scottish Intercollegiate Guidelines Network | [www.sign.ac.uk](http://www.sign.ac.uk) |
| Social Care Institute for Excellence | https://www.scie.org.uk/ |
| South African Cochrane Centre | https://southafrica.cochrane.org/ |
| The International Network of Agencies for Health Technology Assessment (INAHTA) | http://www.inahta.org/members/kce/ |
| UK Cochrane Centre | https://uk.cochrane.org/ |
| United States Preventive Services Task Force | https://www.uspreventiveservicestaskforce.org |
| Workers' Compensation Board Evidence Based Practice Group, Workers' Compensation Board of BC | <https://www.worksafebc.com/en> |
| World Health Organization | https://www.who.int |

1. **Conference proceedings**

**Search Date:** 6-7 February 2019 (Conference Years: 2015-2018)

**Conference Proceedings Searched:** 3

**Search updated:** 3-5 February 2020

| **Conference Name** | **URL** |
| --- | --- |
| International Cochrane Colloquium | <http://www.abstracts.cochrane.org> |
| Health Technology Assessment International (HTAi) | <http://www.htai.org/meetings/annual-meetings/past-annual-meetings.html> |
| Canadian Agency for Drugs and Technologies in Health (CADTH) Symposium | <https://www.cadth.ca/cadth-symposium-archives>  (also used general web searches) |
| Global Evidence Summit | <https://www.globalevidencesummit.org/> |

Note: HTA International could only be searched between 2017-2018 because access to previous years is restricted.

1. **Reference Lists of Relevant Documents**

**Search Date:** Current to March 2020

| **Document** | **Details** |
| --- | --- |
| Lunny et al. 2017 | Toward a comprehensive evidence map of overview of systematic review methods: Paper 1. Syst Rev 2017;6:231. |
| Lunny et al. 2018 | Toward a comprehensive evidence map of overview of systematic review methods: Paper 2. Syst Rev 2018;7:159. |
| Other new included studies | Reviewed the reference lists of included studies |

1. **Contacting Producers of Overviews**

**Date Contacted:** 26 February 2019 (contacted again on 27 March 2019 if no reply on the first attempt)

| **Type of overview producer** | **Number contacted** |
| --- | --- |
| Authors of published overviews* | 100 |
| Managing Editors of Cochrane Review Groups and Fields | 22 |

* Lists of authors were obtained from: Pieper D, Pollock M, Fernandes RM, Büchter RB, Hartling L. Epidemiology and reporting characteristics of overviews of reviews of healthcare interventions published 2012-2016: a systematic review. Preliminary results (reference list of included overviews obtained from the authors). We also e-mailed the managing editors of Cochrane Review Groups that had published at least one overview.

1. **Known Internal (ARCHE) Documents**

N = 3 presentations (1 duplicate)
