# Supplementary material for: Guidance for overviews of reviews continues to accumulate, but important challenges remain: a scoping review
Source: Syst Rev. 2020 Nov 4;9:254. doi: 10.1186/s13643-020-01509-0 (PMC7643411; doi:10.1186/s13643-020-01509-0)
Supplement: Supplementary file 3 — Additional file 3. Studies excluded following full text review. [file 13643_2020_1509_MOESM3_ESM.docx]

**Studies excluded after full text appraisal, N = 178**

**n = 65 not about overviews of reviews of healthcare interventions**

Akl EA, Haddaway NR, Rada G, Lotfi T. Evidence synthesis 2.0: when systematic, scoping, rapid, living, and overviews of reviews come together. Journal of Clinical Epidemiology. 2020; doi: 10.1016/j.jclinepi.2020.01.025.

Alahdab F, Murad MH. Evidence maps: a tool to guide research agenda setting. BMJ Evidence-based Medicine. 2019;24(6):209-11.

Ardern CL, Winters M. Synthesising 'best evidence' in systematic reviews when randomised controlled trials are absent: three tips for authors to add value for clinician readers. British Journal of Sports Medicine. 2018;52(15):948-9.

Bolland MJ, Grey A, Reid IR. Differences in overlapping meta-analyses of vitamin D supplements and falls. The Journal of Clinical Endocrinology and Metabolism. 2014;99(11):4265-72.

Boulesteix AL. Letter to the editor: On reviews and papers on new methods. Briefings in Bioinformatics. 2015;16(2):365-6.

Brunton G, Thomas J, Paraskeva N, Caird J, Rumsey N, editors. Putting the issues on the table: summarising outcomes form reviews of reviews to inform health policy. In: Cochrane Colloquium Quebec City; 2006.

Canadian Task Force on Preventive Health Care. Procedure manual. Ottawa, ON: Canadian Task Force on Preventive Health Care; 2014.

Cheyne S, Lewis S, Askie L, Staub L. Challenges for conducting overviews including observational primary studies. Abstracts of the 26th Cochrane Colloquium, Santiago, Chile. Cochrane Database of Systematic Reviews; 2020.

Economos CD, Sallis JF, Keith NR, Newkirk J. The Active Living Research 2013 Conference: achieving change across sectors: integrating research, policy, and practice. American journal of Health Promotion. 2014;28(3 Suppl):S1-4.

Einhorn TA, Swiontkowski MF. New article type for JBJS Reviews. JBJS Reviews. 2016;4(3).

Gavine A, MacGillivray S, Ross-Davie M, Campbell K, White L, Renfrew M. Maximising the availability and use of high-quality evidence for policymaking: Collaborative, targeted and efficient evidence reviews. Palgrave Communications. 2018;4(1):5.

Gough D, Thomas J, Oliver S. Clarifying differences between review designs and methods. Systematic Reviews. 2012;1(1):28.

Gouveia L. Evento PhD CC-SiTEGI-20 de Julho de 2018. Evento de apresentações do doutoramento em Ciências da Informação, ramo SiTeGI. 2018.

Haas DM. Keeping up with evidence in Cochrane Reviews. Obstetrics and Gynecology. 2020;135(1):4-5.

Hafdahl AR. Article alerts: Introduction and items from 2009, part I. Research Synthesis Methods. 2010;1(1):81-7.

Halén P, Khan KM. Finland's sports physiotherapy conference-athlete training and loading: Helsinki 7-8 June 2019. British Journal of Sports Medicine. 2019;53(3):137-8.

Harder T, Remschmidt C, Haller S, Eckmanns T, Wichmann O. Use of existing systematic reviews for evidence assessments in infectious disease prevention: a comparative case study. Systematic Reviews. 2016;5(1):171.

Hunt H, Hyde C, editors. Constructing an overview of systematic reviews of diagnostic test accuracy. Cochrane Colloquium 2015; Vienna, Austria.

Institut für Qualität und Wirtschaftlichkeit im Gesundheitswesen, IQWiG. Allgemeine Methoden version 6.0. Koln, Germany: IQWiG; 2019.

Juhl CB, Lund H. Do we really need another systematic review? British Journal of Sports Medicine. 2018;52(22):1408-9.

Kastner M, Antony J, Soobiah C, Straus SE, Tricco AC. Conceptual recommendations for selecting the most appropriate knowledge synthesis method to answer research questions related to complex evidence. Journal of Clinical Epidemiology. 2016;73:43-9.

Li D, Wang Z, Shen F, Murad MH, Liu H, editors. Towards a multi-level framework for supporting systematic review—A pilot study. 2014 IEEE International Conference on Bioinformatics and Biomedicine (BIBM); 2014.

Li D, Wang Z, Shen F, Murad MH, Liu H, editors. Towards a multi-level framework for supporting systematic review - A pilot study. 2014 IEEE International Conference on Bioinformatics and Biomedicine. 2-5 November 2014.

Maggio LA, Thomas A, Durning SJ. 31 Knowledge Synthesis. Understanding medical education: evidence, theory, and practice, 3rd edition. Oxford, UK: The Association for the Study of Medical Education (ASME); 2018.

Malterud K. The impact of evidence-based medicine on qualitative metasynthesis: Benefits to be harvested and warnings to be given. Qualitative Health Research. 2019;29(1):7-17.

Manson H. Systematic reviews are not enough: policymakers need a greater variety of synthesized evidence. Journal of Clinical Epidemiology. 2016;73:11-4.

Marshall AP. Systematic reviews, 'systematic reviews' and more: When variation leads to confusion. Australian Critical. 2018;31(5):255-6.

Maynard B, Polanin J, Dell N. Overviews of reviews: Unique challenges and opportunities of synthesising syntheses. Cochrane Colloquium 2017; Cape Town, South Africa.

McKenzie JE, Salanti G, Lewis SC, Altman DG. Meta-analysis and the Cochrane Collaboration: 20 years of the Cochrane Statistical Methods Group. Systematic Reviews. 2013;2(1):80.

Miake-Lye IM, Hempel S, Shanman R, Shekelle PG. What is an evidence map? A systematic review of published evidence maps and their definitions, methods, and products. Systematic Reviews. 2016;5:28.

Misra DP, Ravindran V. Reporting standards in scientific publishing: need, relevance and future perspectives. The Journal of the Royal College of Physicians of Edinburgh. 2019;49(4):269-71.

Moja L, Fernandez del Rio MP, Banzi R, Cusi C, D'Amico R, Liberati A, et al. Multiple systematic reviews: Methods for assessing discordances of results. Internal and Emergency Medicine. 2012;7(6):563-8.

Munn Z, Dias M, Tufanaru C, Porritt K, Stern C, Jordan Z, et al. Adherence of meta-aggregative systematic reviews to reporting standards and methodological guidance: a methodological review protocol. JBI Database of Systematic Reviews and Implementation Reports. 2019;17(4):444-50.

Munn Z, Stern C, Aromataris E, Lockwood C, Jordan Z. What kind of systematic review should I conduct? A proposed typology and guidance for systematic reviewers in the medical and health sciences. BMC Medical Research Methodology. 2018;18(1):5.

National Institute for Health Care Excellence. Developing NICE guidelines: the manual. London, UK: National Institute for Health Care Excellence; 2018.

O'Leary BC, Woodcock P, Kaiser MJ, Pullin AS. Evidence maps and evidence gaps: Evidence review mapping as a method for collating and appraising evidence reviews to inform research and policy. Environmental Evidence. 2017;6(1).

Ongur D. Challenges when conducting complex clinical research. JAMA Psychiatry. 2019;76(10):1017.

Oxman AD, Cook DJ, Guyatt GH, et al. User's guides to the medical literature: VI. How to use an overview. JAMA. 1994;272(17):1367-71.

Oxman AD, Guyatt GH. Validation of an index of the quality of review articles. Journal of Clinical Epidemiology. 1991;44(11):1271-8.

Oxman AD. Helping people make well-informed decisions about health care: old and new challenges to achieving the aim of the Cochrane Collaboration. Systematic Reviews. 2013;2(1):77.

Patrini M, Arienti C, Lazzarini S, Gimigliano F, Kiekens C, Negrini S. Overview of Cochrane Systematic Reviews as a methodological tool to introduce Cochrane evidence in WHO guidelines: the Cochrane Rehabilitation experience. Abstracts of the 26th Cochrane Colloquium, Santiago, Chile. Cochrane Database of Systematic Reviews 2020.

Reilly PA. Issue Overview. Military Medicine. 2020;185(Supplement 1):2.

Reviews covering publications from August 15, 2017 - February 15, 2018. International Journal of Gynecological Cancer. 2018;28(Suppl 1):1-60.

Reviews covering publications from February 15, 2018 - August 15, 2018. International Journal of Gynecological Cancer. 2018;28(Suppl 4):1-57.

Robinson K, Chou R, Berkman N, Newberry S, Fu R, Hartling L, et al. Integrating bodies of evidence: Existing systematic reviews and primary studies. In: Methods guide for effectiveness and comparative effectiveness reviews. Rockville (MD): Agency for Healthcare Research and Quality; 2008.

Robinson K, Whitlock E, O'Neil M, Anderson J, Hartling L, Dryden D, et al. Integration of existing systematic reviews. In: Research white paper (prepared by the Scientific Resource Center under contract no 290-2012-00004-C). Rockville: Agency for Healthcare Research and Quality; 2014.

Robinson KA, Chou R, Berkman ND, Newberry SJ, Fu R, Hartling L, et al. Twelve recommendations for integrating existing systematic reviews into new reviews: EPC guidance. Journal of Clinical Epidemiology. 2016;70:38-44.

Robinson KA, Whitlock EP, Oneil ME, Anderson JK, Hartling L, Dryden DM, et al. Integration of existing systematic reviews into new reviews: identification of guidance needs. Systematic Reviews. 2014;3:60.

Rutter D, Francis J, Coren E, Fisher M. SCIE Research resource 1: SCIE systematic research reviews: guidelines (2nd edition). London, UK: Social Care Institute for Excellence; 2010.

Ryan RE, Kaufman CA, Hill SJ. Building blocks for meta-synthesis: data integration tables for summarising, mapping, and synthesising evidence on interventions for communicating with health consumers. BMC Medical Research Methodology. 2009;9:16-.

Scottish Intercollegiate Guidelines Network. SIGN 50: A guideline developer's handbook. Edinburgh, UK: Scottish Intercollegiate Guidelines Network; 2015.

Scottish Intercollegiate Guidelines Network. SIGN 50: A guideline developer's handbook (SIGN publication no. 50). Edinburgh, Scotland: Scottish Intercollegiate Guidelines Network; 2019.

Snilstveit B, Vojtkova M, Bhavsar A, Stevenson J, Gaarder M. Evidence & Gap Maps: A tool for promoting evidence informed policy and strategic research agendas. Journal of clinical Epidemiology. 2016;79:120-9.

Starr M, Chalmers I, Clarke M, Oxman AD. The origins, evolution, and future of The Cochrane Database of Systematic Reviews. International Journal of Technology Assessment in Health Care. 2009;25(S1):182-95.

Tang LL, Caudy M, Taxman F. A statistical method for synthesizing meta-analyses. Computational and Mathematical Methods in Medicine. 2013;2013:732989.

Tanvejsilp P, Ngorsuraches S. Defining the scope of health technology assessment and types of health economic evaluation. Journal of the Medical Association of Thailand. 2014;97 Suppl 5:S10-6.

The Campbell Collaboration. Campbell systematic reviews: policies and guidelines. Oslo, Norway: The Campbell Collaboration; 2019.

Thorne S. Metasynthetic madness: What kind of monster have we created? Qualitative health Research. 2017;27(1):3-12.

Tillyard G, DeGennaro V, Jr. New methodologies for global health research: improving the knowledge, attitude, and practice survey model through participatory research in Haiti. Qualitative Health Research. 2019;29(9):1277-1286.

Turner T, Green S, Tovey D, McDonald S, Soares-Weiser K, Pestridge C, et al. Producing Cochrane systematic reviews-a qualitative study of current approaches and opportunities for innovation and improvement. Systematic Reviews. 2017;6(1):147.

United States Preventive Services Task Force. US Preventive Services Task Force procedure manual. Rockville, MD: United States Preventive Services Task Force; 2015.

Wagner S, White M, Schultz I, Iverson R, Hsu V, McGuire L, et al., editors. Assessing a systematic review of systematic reviews: developing a criteria. In: Innovation in worker health and safety: Annual Conference, Canadian Association for Research on Work and Health, June 1-2, 2012 Vancouver; 2012.

White CM, Ip S, McPheeters M, Carey TS, Chou R, Lohr KN, et al. Using existing systematic reviews to replace de novo processes in conducting comparative effectiveness reviews. Methods Guide for Effectiveness and Comparative Effectiveness Reviews. Rockville (MD): Agency for Healthcare Research and Quality; 2008.

Whiting P, Davies P, Savovic J, Caldwell D, Churchill R. Evidence to inform the development of ROBIS, a new tool to assess the risk of bias in systematic reviews. 2013. Available from http://www.robis-tool.info

Whitlock EP, Lin JS, Chou R, Shekelle P, Robinson KA. Using existing systematic reviews in complex systematic reviews. Annals of Internal Medicine. 2008;148(10):776-82.

Woodman J, Thomas J, Dickson K. How explicable are differences between reviews that appear to address a similar research question? A review of reviews of physical activity interventions. Systematic Reviews. 2012;1:37.

**n = 72 not intended as methods guidance**

Apostolo J, Bobowicz-Campos E, Holland C, Cooke R, Santana S, Marcucci M, et al. Challenges in conducting an overview of reviews evaluating diagnostic accuracy and predictive ability of frailty screening tools: a practical example. Cochrane Colloquium 2016; Seoul, South Korea.

Bajpai R, Posadzki P, Soljak M, Car J. Managing overlap in overviews of reviews: a cross-sectional survey of the published literature between 2015 and 2017. Cochrane Colloquium 2018; Edinburgh, UK.

Blackwood D. Taking it to the next level: Reviews of systematic reviews. HLA News. 2016 (Winter 2016):13.

Brennan S, McKenzie J, Middleton P, Akl E, Green S, Reid J, et al.. Developing GRADE guidance for overviews of systematic reviews. Cochrane Colloquium 2017; Cape Town, South Africa.

Brennan S, Middleton P, Akl E, Pollock A, Reid J, Mackenzie J, editors. Assessing the certainty of evidence in overviews of reviews: current practice and expert perspectives. Cochrane Colloquium 2018; Edinburgh, UK.

Broderick J, Irving C, Shokraneh F, Ostinelli E, Walshe M, Mockler D, et al. Development of workable solutions to overcome known limitations of overview methodology. Cochrane Colloquium 2018; Edinburgh, UK.

Buchter R, Pieper D, Jerinic P. Overviews of systematic reviews often do not assess methodological quality of included reviews. Cochrane Colloquium 2011; Madrid, Spain.

Buchter RB, Pieper D. Most overviews of Cochrane reviews neglected potential biases from dual authorship. Journal of Clinical Epidemiology. 2016;77:91-4.

Campbell P, Torrens C, MacIntyre A, Pollock A, Maxwell M, Williams J, et al. Can we use overviews to reduce research waste? An example from a population-level intervention overview. Cochrane Colloquium 2017; Cape Town, South Africa.

Chen YF, Hemming K, Chilton PJ, Gupta KK, Altman DG, Lilford RJ. Scientific hypotheses can be tested by comparing the effects of one treatment over many diseases in a systematic review. Journal of Clinical Epidemiology. 2014;67(12):1309-19.

Crick K, Wingert A, Williams K, Fernandes RM, Thomson D, Hartling L. An evaluation of harvest plots to display results of meta-analyses in overviews of reviews: a cross-sectional study. BMC Medical Research Methodology. 2015;15:91.

Dalsbo T, Dahm K, Stromme H. Cochrane overviews of reviews: purpose and quality. Cochrane Colloquium 2018; Edinburgh, UK.

de Guinea AO, Paré G. What literature review type should I conduct? The Routledge Companion to Management Information Systems: Routledge; 2017. p. 93-102.

Dettori JR, Norvell DC. Discordant systematic Reviews: which to believe? Global Spine Journal. 2020;10(2):237-9.

Esposito M. Editorial: Overviews and umbrella reviews. European Journal of Oral Implantology. 2018;11(3):255.

Faggion CM, Jr., Diaz KT. Overview authors rarely defined systematic reviews that are included in their overviews. Journal of Clinical Epidemiology. 2019;109:70-79.

Foisy M, Fernandes R, Hartling L. What guidance is available for researchers conducting overviews of reviews? A scoping review and qualitative metasummary. Cochrane Colloquium 2015; Vienna, Austria.

Garritty C, Stevens A, Hamel C, Golfam M, Hutton B, Wolfe D. Knowledge synthesis in evidence-based medicine. Seminars in Nuclear Medicine. 2019;49(2):136-44.

Hartling L, Dryden D, Vandermeer B, Fernandes R. Generating empirical evidence to support methods for overviews of reviews. Cochrane Colloquium 2013; Quebec, Canada.

Hartling L, Vandermeer B, Fernandes RM. Systematic reviews, overviews of reviews and comparative effectiveness reviews: a discussion of approaches to knowledge synthesis. Evidence-based Child Health: a Cochrane Review Journal. 2014;9(2):486-94.

Hinneburg I. New formats for evidence synthesis. Medizinische Monatsschrift fur Pharmazeuten. 2018;41(1):25-8.

Holly C. Opportunities and challenges in a world of data abundance. JBI Database of Systematic Reviews and Implementation Reports. 2017;15(11):2597-8.

Hunt H, Pollock A, Campbell P, Estcourt L, Brunton G. An introduction to overviews of reviews: planning a relevant research question and objective for an overview. Systematic Reviews. 2018;7(1):39.

Ikezoe T. [Overview]. The Japanese Journal of Clinical Hematology. 2019;60(6):646.

Ioannidis J. Next-generation systematic reviews: prospective meta-analysis, individual-level data, networks and umbrella reviews. British Journal of Sports Medicine. 2017;51(20):1456-8.

James BM, Baker PR, Costello JT, Francis DP. Informing methods for preparing public health overviews of reviews: a comparison of public health overviews with Cochrane overviews published between 1999 and 2014. Cochrane Colloquium 2014. Hyderabad, India.

Jordan Z, Lockwood C, Munn Z, Aromataris E. The updated Joanna Briggs Institute Model of Evidence-Based Healthcare. International Journal of Evidence-based Healthcare. 201;17(1):58-71.

Jordan Z, Munn Z, Aromataris E, Lockwood C. Now that we're here, where are we? The JBI approach to evidence-based healthcare 20 years on. International Journal of Evidence-based Healthcare. 2015;13(3):117-20.

Knafl K, Whittemore R. Top 10 tips for undertaking synthesis research. Research in Nursing and Health. 2017;40(3):189-93.

Kovacs FM, Urrútia G, Alarcón JD. "Overviews’’ should meet the methodological standards of systematic reviews. European Spine Journal. 2014;23(2):480.

Krnic Martinic M, Pieper D, Glatt A, Puljak L. Definition of a systematic review used in overviews of systematic reviews, meta-epidemiological studies and textbooks. BMC Medical Research Methodology. 2019;19(1):203.

Levin RF. Reviews, systematic reviews, overviews:“What’s it all about, Cochrane?”. Research and Theory for Nursing Practice. 2009;23(4):256-8.

Littell J. Conceptual and practical classification of research reviews and other evidence synthesis. Knowledge in Society. 2018;1:104-26.

Lunny C, Brennan S, McDonald S, McKenzie J. An inventory of methods for overviews of systematic reviews of interventions: mapping the evidence for the methods. Cochrane Colloquium 2016; Seoul, South Korea.

Lunny C, Brennan S, Reid J, McDonald S, McKenzie J. Overviews of reviews incompletely report methods for handling overlapping, discordant and problematic data. Journal of Clinical Epidemiology. 2019;118:69-85.

Lunny C, Brennan SE, McDonald S, McKenzie JE. Evidence map of studies evaluating methods for conducting, interpreting and reporting overviews of systematic reviews of interventions: rationale and design. Systematic Reviews. 2016;5:4.

Lunny C, Brennan SE, McDonald S, McKenzie JE. Toward a comprehensive evidence map of overview of systematic review methods: paper 1-purpose, eligibility, search and data extraction. Systematic Reviews. 2017;6(1):231.

Lunny C, Brennan SE, McDonald S, McKenzie JE. Toward a comprehensive evidence map of overview of systematic review methods: paper 2-risk of bias assessment; synthesis, presentation and summary of the findings; and assessment of the certainty of the evidence. Systematic Reviews. 2018;7(1):159.

Lunny C, McKenzie JE, McDonald S. Retrieval of overviews of systematic reviews in MEDLINE was improved by the development of an objectively derived and validated search strategy. Journal of Clinical Epidemiology. 2016;74:107-18.

Mallidou A. Mapping the landscape of knowledge synthesis. Nursing Management. 2014;21(5):30-9.

McKenzie JE, Brennan SE. Overviews of systematic reviews: great promise, greater challenge. Systematic Reviews. 2017;6(1):185.

Merlin T, Tamblyn D, Ellery B. What's in a name? Developing definitions for common health technology assessment product types of the International Network of Agencies for Health Technology Assessment (INAHTA). International Journal of Technology Assessment in Health Care. 2014;30(4):430-7.

Minami Y. [Overview]. The Japanese Journal of Clinical Hematology. 2019;60(6):582-3.

Montori VM, Wilczynski NL, Morgan D, Haynes RB. Optimal search strategies for retrieving systematic reviews from Medline: analytical survey. BMJ. 2005;330(7482):68.

Nagafuji K. [Overview]. The Japanese Journal of Clinical Hematology. 2019;60(6):619.

Papatheodorou S. Author Reply: A critical reflection on the grading of the certainty of evidence in umbrella reviews. European Journal of Epidemiology. 2019;34:891-2.

Paré G, Kitsiou S. Methods for literature reviews. Handbook of eHealth evaluation: An evidence-based approach. Victoria, Canada: University of Victoria; 2017.

Pieper D, Antoine S-L, Morfeld J-C, Mathes T, Eikermann M. Methodological approaches in conducting overviews: current state in HTA agencies. 2014;5(3):187-99.

Pieper D, Büchter RB, Antoine S-L, Eikermann M. Overviews–status quo, potentials and perspectives. Zeitschrift fur Evidenz, Fortbildung und Qualitat im Gesundheitswesen. 2013;107(9):592-6.

Pieper D, Buechter R, Jerinic P, Eikermann M. Overviews of reviews often have limited rigor: a systematic review. Journal of Clinical Epidemiology. 2012;65(12):1267-73.

Pieper D, Pollock M, Fernandes RM, Büchter RB, Hartling L. Epidemiology and reporting characteristics of overviews of reviews of healthcare interventions published 2012–2016: protocol for a systematic review. Systematic Reviews. 2017;6(1):73.

Pieper D, Waltering A, Holstiege J, Buchter RB. Quality ratings of reviews in overviews: a comparison of reviews with and without dual (co-)authorship. Systematic Reviews. 2018;7(1):63.

Pluye P, Hong QN, Bush PL, Vedel I. Opening-up the definition of systematic literature review: the plurality of worldviews, methodologies and methods for reviews and syntheses. Journal of Clinical Epidemiology. 2016;73:2-5.

Polanin JR, Maynard BR, Dell NA. Overviews in education research: a systematic review and analysis. Review of Educational Research. 2017;87(1):172-203.

Pollock A, Brady MC, Farmer SE, Langhorne P, Mead GE, Mehrholz J, et al. The purpose of rating quality of evidence differs in an overview, as compared to guidelines or recommendations. Journal of Clinical Epidemiology. 2016;74:238-40.

Pollock M, Fernandes R, Becker L, Featherstone R, Hartling L, editors. Guidance for conducting overviews of reviews: results from a scoping review and qualitative metasummary. Cochrane Colloquium 2016; Seoul, South Korea.

Pollock M, Fernandes RM, Becker LA, Featherstone R, Hartling L. What guidance is available for researchers conducting overviews of reviews of healthcare interventions? A scoping review and qualitative metasummary. Systematic Reviews. 2016;5(1):190.

Ranson MK, Evans DB. Taking health systems research syntheses to the next level: overviews of systematic reviews. The Cochrane Database of Systematic Reviews. 2017;9:Ed000123.

Rosen M. Systematiska oversikter och metaanalyser blir an viktigare - Men detta verktyg for evidensbaserad medicin kan skarpas och maste granskas kritiskt. Lakartidningen. 2018;115:E66F.

Samnani SS, Vaska M, Ahmed S, Turin TC. Review typology: the basic types of reviews for synthesizing evidence for the purpose of knowledge translation. Journal of the College of Physicians and Surgeons--Pakistan. 2017;27(10):635-41.

Santaguida PL, Keshavarz H, Carlesso LC, Lomotan M, Gross A, MacDermid JC, et al. Suppl 4: a description of the methodology used in an overview of reviews to evaluate evidence on the treatment, harms, diagnosis/classification, prognosis and outcomes used in the management of neck pain. The Open Orthopaedics Journal. 2013;7:461.

Schlesinger S, Schwingshackl L, Neuenschwander M, Barbaresko J. A critical reflection on the grading of the certainty of evidence in umbrella reviews. European Journal of Epidemiology. 2019;34:889-90.

Sheble L. Diffusion of meta-analysis, systematic review, and related research synthesis methods: Patterns, contexts, and impact: The University of North Carolina at Chapel Hill; 2014.

Silva V, Grande AJ, Carvalho AP, Martimbianco AL, Riera R. Overview of systematic reviews - a new type of study. Part II. Sao Paulo medical journal. 2015;133(3):206-17.

Silva V, Grande AJ, Martimbianco ALC, Riera R, Carvalho APV. Overview of systematic reviews-a new type of study, part I: why and for whom? Sao Paulo Medical Journal. 2012;130(6):398-404.

Smith J, Tshoi B, Lee K, editors. PP28 Overview of reviews In HTA - opportunities and challenges in supporting. HTAi 2018 Annual Meeting: Strengthening the evidence-to-action connection; Vancouver, Canada.

Solmi M, Correll CU, Carvalho AF, Ioannidis JPA. The role of meta-analyses and umbrella reviews in assessing the harms of psychotropic medications: Beyond qualitative synthesis. Epidemiology and Psychiatric Sciences. 2018;27(6):537-42.

Taylor S, Pinnock H, Pearce G. Conducting meta-reviews. Presentation for the Cochrane Collaboration (unpublished; received from authors).

Thomson D. Evidence synthesis in child health: overviews of reviews. Evidence-based Child Health: A Cochrane Review Journal. 2014;9(1):1-2.

Torres A, Tennant B, Ribeiro-Lucas I, Vaux-Bjerke A, Piercy K, Bloodgood B. Umbrella and systematic review methodology to support the 2018 physical activity guidelines advisory committee. Journal of Physical Activity and Health. 2018;15(11):805-10.

Whittemore R, Chao A, Jang M, Minges KE, Park C. Methods for knowledge synthesis: an overview. Journal of Critical Care. 2014;43(5):453-61.

高洪阳, 赵阳, 高蕊, 李博. Cochrane Overview 的研究现状及制作方法调查. 中国循证医学杂志. 2014;14(12):1514-9.

**n = 8 provide guidance for reporting, not conducting overviews**

Bougioukas KI, Bouras E, Apostolidou-Kiouti F, Kokkali S, Arvanitidou M, Haidich AB. Reporting guidelines on how to write a complete and transparent abstract for overviews of systematic reviews of health care interventions. Journal of Clinical Epidemiology. 2019;106:70-9.

Bougioukas KI, Liakos A, Tsapas A, Ntzani E, Haidich AB. Preferred reporting items for overviews of systematic reviews including harms checklist: a pilot tool to be used for balanced reporting of benefits and harms. Journal of Clinical Epidemiology. 2018;93:9-24.

Jia P, Chen J, Zhang L, Zhao P, Zhang M. Challenges and considerations in assessing the reporting quality of systematic reviews in overviews using PRISMA. Cochrane Colloquium 2015; Vienna, Austria.

Li L, Tian J, Tian H, Sun R, Liu Y, Yang K. Quality and transparency of overviews of systematic reviews. Journal of Evidence-based Medicine. 2012;5(3):166-73.

Lunny C, McDonald S, Brennan S, McKenzie J. Assessment of the completeness of reporting of methods in overviews of systematic reviews. Cochrane Colloquium 2018; Edinburgh, UK.

Pieper D, Koensgen N, Breuing J, Ge L, Wegewitz U. How is AMSTAR applied by authors - a call for better reporting. BMC medical research methodology. 2018;18(1):56.

Posadzki P, editor Standards for Reporting of Overviews of Reviews and Umbrella Reviews (STROVI) statement. Cochrane Colloquium 2017; Cape Town, South Africa.

Singh JP. Development of the metareview assessment of reporting quality (MARQ) checklist. Revista de la Facultad de Medicina. 2012;60(4):325-32.

**n = 9 overviews of reviews**

Australian Government National Health and Medical Research Council. NHMRC report on the evidence: Promoting social and emotional development and wellbeing of infants in pregnancy and the first year of life. Australia: National Health and Medical Research Council, Government of Australia; 2017.

Cooney L, Loke YK, Golder S, Kirkham J, Jorgensen A, Sinha I, et al. Overview of systematic reviews of therapeutic ranges: methodologies and recommendations for practice. BMC Medical Research Methodology. 2017;17(1):84.

Gardner J, Lau F, Kuziemsky C, Price M. A review on systematic reviews of health information system studies. Journal of the American Medical Informatics Association. 2010;17(6):637-45.

Genuneit J, Seibold AM, Apfelbacher CJ, Konstantinou GN, Koplin JJ, La Grutta S, et al. Overview of systematic reviews in allergy epidemiology. Allergy. 2017;72(6):849-56.

Hadnorntun P, Bangpan M, Taychakhoonavudh S, Thavorn K, Ng S, Chaiyakunapruk N. The challenge of assessing quality and synthesising evidence: Lessons from an overview of systematic reviews of economic evaluations of vaccination programmes. Cochrane Colloquium 2017; Cape Town, South Africa.

O'Mara A, Jamal F, Parry W, Lorenc T, Cooper C. Guidelines for conducting and reporting reviews of reviews: dealing with topic relevances and double-counting. 2011 Cochrane Colloquium; Madrid, Spain.

Plonsky L, Gonulal T. Methodological synthesis in quantitative L2 research: A review of reviews and a case study of exploratory factor analysis. Language Learning. 2015;65(S1):9-36.

Rehfuess EA, Stratil JM, Scheel IB, Portela A, Norris SL, Baltussen R. The WHO-INTEGRATE evidence to decision framework version 1.0: integrating WHO norms and values and a complexity perspective. BMJ Global Health. 2019;4(Suppl 1):e000844.

Riva N, Puljak L, Moja L, Ageno W, Schünemann H, Magrini N, et al. Multiple overlapping systematic reviews facilitate the origin of disputes: the case of thrombolytic therapy for pulmonary embolism. Journal of Clinical Epidemiology. 2018;97:1-13.

**n = 12 duplicates**

Aromataris E, Fernandez R, Godfrey CM, Holly C, Khalil H, Tungpunkom P. Summarizing systematic reviews: methodological development, conduct and reporting of an umbrella review approach. International Journal of Evidence-based Healthcare. 2015;13(3):132-40.

Chen T-T, Tu Y-K. Statistical models for overviews of reviews. In: Biondi-Zoccai G (editor). Umbrella Reviews. Cham, Switzerland: Springer; 2016, p. 137-53.

Giannakou K, Galanis P. Umbrella reviews in clinical research. Archives of Hellenic Medicine. 2020;37(1):129-34.

Hartling L, Fernandes R, Becker L, Foisy M, editors. Comparing multiple treatments: an introduction to overviews of reviews. Cochrane Colloquium 2015; Vienna, Austria.

Lewin S, Oxman A, Ciapponi A, Herrera C, Opiyo N, Pantoja T, et al., editors. Novel approaches to conducting overviews of reviews: Lessons from four overviews of health systems interventions. Cochrane Colloquium 2017; Cape Town, South Africa.

Lewis R, Hendry M, Din N, Stanciu MA, Nafees S, Hendry A, et al. Pragmatic methods for reviewing exceptionally large bodies of evidence. Systematic Reviews. 2019;171(8).

Ortega A, Lopez-Briz E, Fraga-Fuentes MD. From Qualitative Reviews to Umbrella Reviews. In: Biondi-Zoccai G (editor). Umbrella Reviews. Cham, Switzerland: Springer; 2016, p. 21-41.

Papageorgiou SN, Biondi-Zoccai G. Designing the Review. In: Biondi-Zoccai G (editor). Umbrella Reviews. Cham, Switzerland: Springer; 2016, p. 57-80.

Pieper D, Li L, Büchter RB. Avenues for further research In: Biondi-Zoccai G (editor). Umbrella Reviews. Cham, Switzerland: Springer; 2016, p. 373-88.

Pollock M, Fernandes R, Brennan S, Hartling L, Becker L, Thomson D, editors. When, why, and how to conduct a Cochrane overview of reviews. Cochrane Colloquium 2016; Seoul, South Korea.

Pollock M. Integrating evidence across reviews: an introduction to overviews of reviews. Presentation for the Canadian Agency for Drugs and Technologies in Health 2017 (unplublished, received from authors).

Tsagris M, Fragkos KC. Umbrella reviews, overviews of reviews, and meta-epidemiologic studies: similarities and differences In: Biondi-Zoccai G (editor). Umbrella Reviews. Cham, Switzerland: Springer; 2016, p. 43-54.

**n = 2 documents could not be retrieved**

Larsen CG. [Are systematic reviews and meta-analyses a dying research discipline?]. Ugeskrift for laeger. 2015;177(18):887.

Dickson K, Sutcliffe K, Rees R, Thomas J. Evidence for Policy and Practice Information and Co-ordinating Centre (EPPI-Centre), Social Science Research Unit, UCL Institute of Education, London, UK. 2015.

**n = 4 documents updated since 2016 scoping review (replaced)**

Aromataris E, Fernandez R, Godfrey C, Holly C, Khalil H, Tungpunkom P, editors. The Joanna Briggs Institute reviewers' manual 2014: methodology for JBI umbrella reviews. University of Adelaide: Joanna Briggs Institute; 2014.

Aromataris E, Fernandez R, Godfrey CM, Holly C, Khalil H, Tungpunkom P. Summarizing systematic reviews: methodological development, conduct and reporting of an umbrella review approach. Int J Evid Based Healthc. 2015;13(3):132-40.

Becker LA, Oxman AD. Chapter 22: overviews of reviews. In: Higgins JPT, Green S, editors. Cochrane handbook for systematic reviews of interventions (version 5.1.0). The Cochrane Collaboration; 2011.

Norwegian Knowledge Centre for the Health Services. 2: Vare ulike produkter [2: Our various products]. In: Slik oppsummerer vi forskning: handbok for Nasjonalt kunnskapssenter for helsetjenesten (reviderte utg 3.2) [How we summarize research: handbook for Norwegian Knowledge Centre for the Health Services (revised edition 3.2)]. Oslo: Norwegian Centre for the Health Services; 2013.

**n = 5 abstracts for which a full document is included (or previously excluded for another reason)**

Aromataris E, Fernandes R, Godfrey C, Holly C, Khalil H, Tungpunkom P, editors. Umbrella reviews: development and reporting of an approach to summarize systematic reviews. Cochrane Colloquium 2016; Seoul, South Korea.

Buchter R, Holstiege J, Waltering A, Pieper D, editors. Quality ratings of reviews in overviews: a comparison of reviews with and without dual (co-)authorship. Cochrane Colloquium 2017; Cape Town, South Africa.

Buchter R, Pieper D, editors. How do authors of Cochrane Overviews deal with conflicts of interest relating to their own systematic reviews? Cochrane Colloquium 2015; Vienna, Austria.

Lunny C, Brennan S, Steve M, McKenzie J, editors. Mapping methods to deal with commonly encountered scenarios in overviews of reviews. Cochrane Colloquium 2018; Edinburgh, UK.

Lunny C, McKenzie J, McDonald S, Brennan S, editors. An evidence map of studies evaluating methods for conducting, interpreting, and reporting overviews of systematic reviews. Cochrane Colloquium 2015; Vienna, Austria.
