# Supplementary material for: Guidance for overviews of reviews continues to accumulate, but important challenges remain: a scoping review
Source: Syst Rev. 2020 Nov 4;9:254. doi: 10.1186/s13643-020-01509-0 (PMC7643411; doi:10.1186/s13643-020-01509-0)
Supplement: Supplementary file 4 — Additional file 4. Documents included in the scoping review. [file 13643_2020_1509_MOESM4_ESM.docx]

**Documents that contain explicit methodological guidance for conducting overviews (59 documents produced by 24 research groups)**

**Alberta Research Centre for Health Evidence, University of Alberta (ARCHE)**

1. Pollock, M. Advancing methods for overviews of reviews of healthcare interventions [dissertation]. Edmonton, Canada: University of Alberta; 2017. [Available online: <https://era.library.ualberta.ca/items/df8da21e-ae75-41c5-8d88-8d6aeba0cfb2>. Accessed 1 June 2020.]
2. Pollock M, Fernandes RM, Hartling L. Evaluation of AMSTAR to assess the methodological quality of systematic reviews in overviews of reviews of healthcare interventions. BMC Med Res Methodol 2017;17(1):48.
3. Pollock M, Fernandes RM, Newton AS, Scott SD, Hartling L. A decision tool to help researchers make decisions about including systematic reviews in overviews of reviews of healthcare interventions. Syst Rev 2019;8(1):29.

**Belgian Health Care Knowledge Centre (KCE)**

1. Mistiaen P, Leroy R, van de Voorde C, Stordeur S, Van den Heede K. HSR Process notes: literature review and international comparison. Brussels, Belgium: Belgian Health Care Knowledge Centre; 2016.

**Central South University (CSU)**

1. Liu H, Dehua H, Huaiqiong Y. Umbrella reviews—A new method related to evidence-based medical analysis. Chinese J Epidemiol. 2020;41(2):261-6.

**Cochrane Child Health Field (CHF)**

1. Becker LA, Thomson D, Caldwell D. Addressing multiple treatments: i - Cochrane overviews. Abstracts of the Joint Colloquium of The Cochrane and Campbell Collaborations, 18-22 Oct 2010. Keystone, USA: John Wiley & Sons; 2010. [Available online: http://www.cmim. cochrane.org/keystone-2010. Accessed 30 Nov 2015.]
2. Cochrane Child Health. Getting started on an overview of reviews. n.d. [Unpublished.]
3. Cochrane Child Health. Notes on the process of preparing an umbrella review for Evidence-Based Child Health. n.d. [Unpublished.]
4. Foisy M, Thomson D, Dryden DM, Fernandes RM, Hartling L. Conducting overviews of reviews: lessons learned since 2006. Abstracts of the 22nd Cochrane Colloquium, 21-26 Sep 2014. Hyderabad, India: John Wiley & Sons; 2014.
5. Foisy M, Thomson D, Dryden DM, Hartling L. Overviews of reviews: a new publication type and an emerging method of knowledge synthesis. Connecting Through Research Pediatric Research Day, 30 May 2014. Edmonton, Canada.
6. Foisy M, Thomson D, Dryden DM, Hartling L. Overviews of reviews: a new publication type and an emerging method of knowledge synthesis. Women and Children's Health Research Institute Research Day, 12 Nov 2014. Edmonton, Canada.
7. Foisy M. An introduction to overviews of reviews (umbrella reviews). Northern Alberta Health Libraries Association Leading Edge Symposium, 26 Nov 2014. Edmonton, Canada.
8. Hartling L, Fernandes R, Becker L, Foisy M. Comparing multiple treatments: an introduction to overviews of reviews. Abstracts of the 23rd Cochrane Colloquium, 03-07 Oct 2015. Vienna, Austria: John Wiley & Sons; 2015.
9. Thomson D, Becker LA, Foisy M. A primer to Cochrane overviews of reviews. Cochrane Canada Live Webinars, 06 Jun 2011. Online. [Available online: https://www.youtube.com/watch?v=HzSPAvpWpl8. Accessed 1 June 2020.]
10. Thomson D, Foisy M, Hartling L. Overviews of reviews: what they are, what they aren't and how and when to do one. Cochrane Canada Live Webinars, 05 Dec 2013. Online (no longer available to view as of 2020. [See: https://canada.cochrane.org/2013-webinars#overviews. Accessed 1 June 2020.]
11. Thomson D, Russell K, Becker LA, Klassen T, Hartling L. The evolution of a new publication type: steps and challenges of producing overviews of reviews. Res Synth Meth. 2010;1(3-4):198-211.

**Cochrane Chile (CChile)**

1. Pérez-Bracchiglione J, Niño de Guzmán E, Roqué Figuls M, Urrútia G. Graphical representation of overlap degree of primary studies in systematic reviews included in overviews. Abstracts of the 26th Cochrane Colloquium, Santiago, Chile. Cochrane Database Syst Rev 2019;1(Suppl 1):151-2. [Available online: https://colloquium2019.cochrane.org/abstracts/graphical-representation-overlap-degree-primary-studies-systematic-reviews-included. Accessed 1 June 2020.]

**Cochrane Comparing Multiple Interventions Methods Group (CMIMG)**

A6. Becker LA, Thomson D, Caldwell D. Addressing multiple treatments: i - Cochrane overviews. Abstracts of the Joint Colloquium of The Cochrane and Campbell Collaborations, 18-22 Oct 2010. Keystone, USA: John Wiley & Sons; 2010. (duplicate from CHF)

A13. Hartling L, Fernandes R, Becker L, Foisy M. Comparing multiple treatments: an introduction to overviews of reviews. Abstracts of the 23rd Cochrane Colloquium, 03-07 Oct 2015. Vienna, Austria: John Wiley & Sons; 2015. (duplicate from CHF)

A14. Thomson D, Becker LA, Foisy M. A primer to Cochrane overviews of reviews. Cochrane Canada Live Webinars, 06 Jun 2011. Online. [Available online: https://www.youtube.com/watch?v=HzSPAvpWpl8. Accessed 1 June 2020.] (duplicate from CHF)

1. Becker L, Caldwell D, Higgins J, Li T, Salanti G, Schmid C. Comparing multiple interventions in Cochrane reviews. In: Comparing multiple interventions in Cochrane reviews. Cochrane Comparing Multiple Interventions Methods Group. 2013. [Available online: https://methods.cochrane.org/cmi/sites/methods.cochrane.org.cmi/files/public/uploads/Comparing%20Multiple%20Interventions%20in%20Cochrane%20Reviews%20-%202003%2003%2023.pdf. Accessed 1 June 2020.]
2. Becker L, Caldwell D. Comparing multiple treatments: overviews versus intervention reviews. Abstracts of the 19th Cochrane Colloquium, 19-22 Oct 2011. Madrid, Spain: John Wiley & Sons; 2011.
3. Becker LA, Caldwell D, Salanti G, Li T. Editorial considerations for reviews that compare multiple interventions. In: Editorial considerations for reviews that compare multiple interventions. Cochrane Comparing Multiple Interventions Methods Group. 2013. Available online: https://methods.cochrane.org/cmi/editorial-considerations-reviews-compare-multiple-interventions. Accessed 1 June 2020.]
4. Becker LA, Li T, Caldwell D. Comparing multiple treatments 1: overview or intervention review. Abstracts of the 20th Cochrane Colloquium, 30 Sep-03 Oct 2012. Auckland, New Zealand: John Wiley & Sons; 2012.
5. Caldwell DM, Welton NJ, Ades AE. Mixed treatment comparison analysis provides internally coherent treatment effect estimates based on overviews of reviews and can reveal inconsistency. J Clin Epidemiol. 2010;63(8):875-82.
6. Hartling L, Brennan S, Pieper D, Fernandes R, Pollock M. When, why and how to conduct a Cochrane overview of reviews. 26^th^ Cochrane Colloquium, 2018, Edinburgh, Scotland. Cochrane Database Syst Rev. 2018;9(Suppl 1):9. [Available online: https://doi.org/10.1002/14651858.CD201801. Accessed 1 June 2020.]
7. Hartling L, Pollock M, Fernandes R, Brennan S. Integrating evidence across reviews: an introduction to overviews of reviews. Presented to the Canadian Agency for Drugs and Technologies in Health, 2 March 2017.
8. Hartling L. Quality appraisal in overviews. Health Technology Assessment International Annual Meeting, 2018. Vancouver, Canada; 2018. [Available online: https://htai.org/wp-content/uploads/2018/08/AM18_Abstract-Book.pdf. Accessed 1 June 2020.]
9. Li T. Comparing multiple treatments: − intervention review or overview − part 1. Abstracts of the 21st Cochrane Colloquium, 19-23 Sep 2013. Quebec City, Canada: John Wiley & Sons; 2013. [Available online: https://methods.cochrane.org/sites/methods.cochrane.org.cmi/files/public/uploads/NMA%20Quebec%20City%20Workshop%201%20-%202013%2009%2020.pdf. Accessed 1 June 2020.]
10. Pollock M, Fernandes RM, Becker LA, Pieper D, Hartling L. Chapter V: Overviews of reviews. In: Higgins JPT, Thomas J, Chandler J, Cumpstone MS, Li T, Page MJ, Welch V (editors). Cochrane handbook for systematic reviews of interventions, version 6.0 (updated March 2020). Cochrane, 2020. [Available online: www.training.cochrane.org/handbook. Accessed 1 June 2020.]
11. Pollock M, Hartling L. Overviews of systematic reviews: an emerging method in health technology assessment. Canadian Agency for Drugs and Technologies in Health Symposium, 2019. Edmonton, Canada.
12. Li T, Becker LA. Comparing multiple treatments 1: intervention review or overview? Abstracts of the 22nd Cochrane Colloquium, 21-26 Sep 2014. Hyderabad, India: John Wiley & Sons; 2014.
13. Salanti G, Becker LA, Caldwell D, Higgins J, Li T, Schmid C. Evolution of Cochrane intervention reviews and overviews of reviews to better accommodate comparisons among multiple interventions. In: Evolution of Cochrane intervention reviews and overviews of reviews to better accommodate comparisons among multiple interventions. Cochrane Comparing Multiple Interventions Methods Group. 2011. [Available online: https://methods.cochrane.org/sites/methods.cochrane.org.cmi/files/public/uploads/CMIMG%20summary%20of%20meeting%20Milan%20March%202011.pdf. Accessed 1 June 2020.]
14. Agency for Healthcare Research and Quality Evidence-based Practice Centre Program Working Group 3: integrating bodies of evidence: systematic reviews and individual studies. Interview transcript: Lorne Becker. 2014. [Unpublished.]

1. Cochrane Comparing Multiple Interventions Methods Group. Comparing Multiple Interventions Methods Group meeting minutes. In: Paris meeting - comparing multiple interventions in Cochrane reviews. Cochrane Comparing Multiple Interventions Methods Group. 2012. Available online: http://www.cmim. cochrane.org/Paris-2012 (Follow link saying "Minutes of the Meeting"). Accessed 1 June 2020.]
2. Cochrane Comparing Multiple Interventions Methods Group. Editorial decision tree for overviews. In: Comparing multiple interventions in Cochrane reviews. Cochrane Comparing Multiple Interventions Methods Group. 2013. [Available online; https://methods.cochrane.org/sites/methods.cochrane.org.cmi/files/public/uploads/DecisionChart.pdf. Accessed 1 June 2020.]
3. Cochrane Comparing Multiple Interventions Methods Group. Methods innovation fund - stream 1. 2013. [Available online: https://methods.cochrane.org/cmi/methods-innovation-fund-stream-1. Accessed 1 June 2020.]
4. Cochrane Comparing Multiple Interventions Methods Group. Multiple intervention reviews: reflections from CoEds discussions this week. In: Paris meeting - comparing multiple interventions in Cochrane reviews. Cochrane Comparing Multiple Interventions Methods Group. 2012. Available online: http://www.cmim. cochrane.org/Paris-2012 (Follow link saying "Powerpoint summary of CoEds discussion"). Accessed 1 June 2020.]
5. Cochrane Comparing Multiple Interventions Methods Group. Review type and methodological considerations - background paper for the first part of the Paris CMIMG discussion. In: Paris meeting - comparing multiple interventions in Cochrane reviews. Cochrane Comparing Multiple Interventions Methods Group. 2012. [Available online: http://www.cmim. cochrane.org/Paris-2012 (Follow link saying "Background paper"). Accessed 1 June 2020.]

**Cochrane Effective Practice and Organisation of Care Group (EPOC)**

1. Worswick J, Wayne SC. Methodology of meta-synthesis: overviews of systematic reviews. Canadian Agency for Drugs and Technology in Health Symposium, 03-05 Apr 2011. Vancouver, Canada.

**Cochrane Public Health Group (CPH)**

1. Baker PR, Costello JT, Dobbins M, Waters EB. The benefits and challenges of conducting an overview of systematic reviews in public health: a focus on physical activity. J Public Health (Oxf). 2014;36(3):517-21.

**Duke University (DukeU)**

1. Cooper H, Koenka AC. The overview of reviews: unique challenges and opportunities when research syntheses are the principal elements of new integrative scholarship. Am Psychol. 2012;67(6):446-62.

**Evidence for Policy and Practice Information and Co-ordinating Centre (EPPI)**

1. Caird J, Sutcliffe K, Kwan I, Dickson K, Thomas J. Mediating policy-relevant evidence at speed: are systematic reviews of systematic reviews a useful approach? Evid Policy. 2015;11(1):81-97.
2. Thomas J. What should we expect from overviews? 23rd Cochrane Colloquium, Overviews of Systematic Reviews Post-Colloquium Symposium, 08 Oct 2015. Vienna, Austria.

**Glasgow Caledonian University (GCU)**

1. Pollock A, van Wijck F. Cochrane overviews: how can we optimise their impact on evidence-based rehabilitation? Eur J Phys rehabil Med 2019;55(3):395-410.

**Harvard University and the Cyprus University of Technology (HarvU)**

1. Papatheodorou S. Umbrella reviews: what they are and why we need them. Eur J Epidemiol 2019;34(6):543-546.

**Joanna Briggs Institute Umbrella Methodology Group (JBI)**

1. Aromataris E, Fernandez R, Godfrey C, Holly C, Khalil H, Tungpunkom P, editors. Chapter 10: Umbrella reviews. In: The Joanna Briggs Institute reviewers' manual 2017. University of Adelaide: Joanna Briggs Institute; 2017. [Available online: https://wiki.joannabriggs.org/display/MANUAL/JBI+Reviewer%27s+Manual. Accessed 1 June 2020.]
2. Aromataris E, Fernandez R, Godfrey CM, Holly C, Khalil H, Tungpunkom P. Summarizing systematic reviews: methodological development, conduct and reporting of an umbrella review approach. Int J Evid Based Healthc. 2015;13(3):132-40.
3. Joanna Briggs Institute Umbrella Review Methods Group. Umbrella review - systematic review methods group progress report. 2013. [Unpublished.]

**King’s College London (KCL)**

1. Fusar-Poli P, Radua J. Ten simple rules for conduction umbrella reviews. Evid Based Mental Health 2018;21(3):95-100.

**Northeast Institute of Evidence Synthesis and Translation at Rutgers School of Nursing (NEST)**

1. Holly C. Umbrella reviews. In: Holly C, Salmond S, Saimbert M (editors). Comprehensive Systematic Review for Advanced Practice Nursing. New York, NY: Springer Publishing; 2016.

**Norwegian Knowledge Centre for the Health Services (NOKC)**

1. Norwegian Knowledge Centre for the Health Services. Slik oppsummerer vi forskning. Håndbok for Nasjonalt kunnskapssenter for helsetjenesten. 4. reviderte utg. [How we summarize research: handbook for Norwegian Knowledge Centre for the Health Services (revised edition 4)]. Oslo: Norwegian Centre for the Health Services; 2015.

**Sapienza University of Rome (SUR)**

1. Biondi-Zoccai G (editor). Umbrella reviews: evidence synthesis with overviews of reviews and meta-epidemiologic studies, Part II: Sound design, conduct, and reporting. Cham, Switzerland: Springer International Publishing; 2016.

**Trinity College Dublin (TCD)**

1. Smith V, Devane D, Begley CM, Clarke M. Methodology in conducting a systematic review of systematic reviews of healthcare interventions. BMC Med Res Methodol. 2011;11(1):15.

**University of Birmingham (UBirm)**

1. Hemming K, Bowater RJ, Lilford RJ. Pooling systematic reviews of systematic reviews: a Bayesian panoramic meta-analysis. Stat Med. 2012;31(3):201-16.

**University of Connecticut (UConn)**

1. Hennessy EA, Johnson BT, Keenan C. Best practice guidelines and essential methodological steps to conduct rigorous and systematic meta-reviews. Appl Psychol Health Well Being 2019;11(3):353-81.
2. Hennessy EA, Johnson BT. Examining overlap of included studies in meta-reviews: guidance for using the corrected covered area index. Res Synth Methods 2019;11(1). doi: 10.1002/jrsm.1390

**University of Cyprus (UCyp)**

1. Giannakou K, Galanis P. Umbrella reviews in clinical research. Hellenic Medicine Archives 2020;37(1):129-34.

**University of Oxford (UOx)**

1. Ballard M, Montgomery P. Risk of bias in overviews of reviews: a scoping review of methodological guidance and four-item checklist. Res Synth Methods 2017;8(1):92-108.

**Western Journal of Nursing Research (WJNR)**

1. Conn VS, Coon Sells TG. WJNR welcomes umbrella reviews. West J Nurs Res. 2014;36(2):147-51.

**Witten/Herdecke University (WHU)**

1. Pieper D, Antoine SL, Mathes T, Neugebauer EA, Eikermann M. Systematic review finds overlapping reviews were not mentioned in every other overview. J Clin Epidemiol. 2014;67(4):368-75.
2. Pieper D, Antoine SL, Neugebauer EA, Eikermann M. Up-to-dateness of reviews is often neglected in overviews: a systematic review. J Clin Epidemiol. 2014;67(4):1302-8.

**Documents that describe an author team's experience conducting one or more published overviews (20 documents produced by 16 research groups)**

**Alberta Research Centre for Health Evidence, University of Alberta (ARCHE)**

1. Pollock M, Fernandes RM, Newton AS, Scott SD, Hartling L. The impact of different inclusion decisions on the comprehensiveness and complexity of overviews of reviews of healthcare interventions. Syst Rev 2019;8(1):18.

**Cochrane Child Health Field (CHF)**

1. Foisy M, Becker LA, Chalmers JR, Boyle RJ, Simpson EL, Williams HC. Mixing with the ‘unclean’: including non-Cochrane reviews alongside Cochrane reviews in overviews of reviews. Abstracts of the 19th Cochrane Colloquium, 19-22 Oct 2011. Madrid, Spain: John Wiley & Sons; 2011.
2. Thomson D, Foisy M, Oleszczuk M, Wingert A, Chisholm A, Hartling L. Overview of reviews in child health: evidence synthesis and the knowledge base for a specific population. Evid Based Child Health. 2013;8(1):3-10.

**Cochrane Chile (CChile)**

A17. Pérez-Bracchiglione J, Niño de Guzmán E, Roqué Figuls M, Urrútia G. Graphical representation of overlap degree of primary studies in systematic reviews included in overviews. Abstracts of the 26th Cochrane Colloquium, Santiago, Chile. Cochrane Database Syst Rev 2020;1(Suppl 1):151-2. [Available online: https://colloquium2019.cochrane.org/abstracts/graphical-representation-overlap-degree-primary-studies-systematic-reviews-included. Accessed 1 June 2020.]

**Cochrane Consumers and Communication Review Group (CCG)**

1. Ryan RE, Kaufman CA, Hill SJ. Building blocks for meta-synthesis: data integration tables for summarising, mapping, and synthesising evidence on interventions for communicating with health consumers. BMC Med Res Methodol. 2009;9:16.

**Cochrane Effective Practive and Organisation of Care Group (EPOC)**

1. Flodgren GS, Shepperd S, Eccles, M. Challenges facing reviewers preparing overviews of reviews. Abstracts of the 19th Cochrane Colloquium, 19-22 Oct 2011. Madrid, Spain: John Wiley & Sons; 2011.
2. Pantoja T, Opiyo N, Ciaponni A, Herrera C, Lewin S, Oxman A, et al. Strategies for improving health systems in low-income countries: lessons learnt from four overviews of systematic reviews of health systems interventions. Abstracts of the 23rd Cochrane Colloquium, 03-07 Oct 2015. Vienna, Austria: John Wiley & Sons; 2015.
3. Lewin S, Ciapponi A, Herrera C, Opiyo N, Oxman A, Pantoja T, et al. Novel approaches to conducting overviews of reviews: lessons from four overviews of health systems interventions. IECS Cochrane Centre Congress 2017. [Available online: https://www.iecs.org.ar/presentaciones/. Accessed 1 June 2020.]

**Cochrane Muskuloskeletal Group (CMG)**

1. Tanjong Ghogomu E, Maxwell L, Singh J, Christensen R, Wells G, Buchbinder R, et al. Overcoming methodological challenges associated with network meta-analysis: the experience of the Musculoskeletal Group. Abstracts of the 19th Cochrane Colloquium, 19-22 Oct 2011. Madrid, Spain: John Wiley & Sons; 2011.

**Cochrane Stroke Group (CSG)**

1. Pollock A, Farmer SE, Brady MC, Langhorne P, Mead GE, Mehrholz J, et al. Completing the first Cochrane overview of stroke reviews: experiences of the Cochrane Stroke Group. Abstracts of the 23rd Cochrane Colloquium, 03-07 Oct 2015. Vienna, Austria: John Wiley & Sons; 2015.

**Dutch Cochrane Centre (DCC)**

1. Kramer SFL, Langendam M, Elbers R, Scholten R, Hooft L. Preparing an overview of reviews: lessons learned. Abstracts of the 17th Cochrane Colloquium, 11-14 Oct 2009. Singapore, Singapore: John Wiley & Sons; 2009.

**Glasgow Caledonian University (GCU)**

1. Pollock A, Campbell P, Brunton G, Hunt H, Estcourt L. Selecting and implementing overview methods: implications from five exemplar overviews. Syst Rev. 2017;6(1):145.
2. Pollock A, Farmer SE, Brady MC, Longhorne P, Mead GE, Mehrholz J. An algorithm was developed to assign GRADE levels of evidence to comparisons within systematic reviews. J Clin Epidemiol 2016;70:106-10.

**King’s College London (KCL)**

A47. Fusar-Poli P, Radua J. Ten simple rules for conduction umbrella reviews. Evid Based Mental Health 2018;21(3):95-100.

**Ludwig Boltzmann Institute for Health Technology Assessment (LBI)**

1. Piso B, Semlitsch T, Reinsperger I, Breuer J, Kaminski-Hartenthaler A, Kien C, et al. Praxiserfahrungen mit overviews of reviews - wertvolle entscheidungsunterstützung oder wissenschaftliche fingerübung? [Practical experience with overviews of reviews - valuable decision aid or academic exercise?]. Z Evid Fortbild Qual Gesundhwes. 2015;109(4-5):300-8.

**Pontifical Xavierian University (PXU)**

1. Rojas ML, Lozano J, Sola I, Bonfill X. Incorporating the GRADE approach in overviews of systematic reviews: an example from an overview in neonatal respiratory care. Abstracts of the 19th Cochrane Colloquium, 19-22 Oct 2011. Madrid, Spain: John Wiley & Sons; 2011.

**Robinson Research Institute; University of Adelaide (RBI)**

1. Shepherd E, Middleton P, Crowther C. Challenges of overviews of reviews and how to overcome them, informed by a public health overview. 24^th^ Cochrane Colloquium, 2016. Seoul, South Korea. [Available online: http://2016.colloquium.cochrane.org/abstracts-e-book, p.6. Accessed 1 June 2020.]

**University of Auckland (UAuck)**

1. Bofill Rodriguez M, Jordan C, Wise M, Lethaby A, Farguhar C. How difficult is it to do an overview of Cochrane reviews? Could core outcomes help? Abstracts of the 26th Cochrane Colloquium, Santiago, Chile. Cochrane Database Syst Rev 2020;1(Suppl 1):257-8. [Available online: https://colloquium2019.cochrane.org/abstracts/how-hard-do-overview-cochrane-reviews-could-core-outcomes-help. Accessed 1 June 2020.]

**University of Calgary (UCalg)**

1. Misfeldt R, Hepp S. Research design of overview of reviews: incentive for health care providers. SAGE research methods cases. London, UK: SAGE publications; 2017.

**University of Dundee (UDun)**

1. Elliott L, Crombie IK, Irvine L, Cantrell J, Taylor J. The effectiveness of public health nursing: the problems and solutions in carrying out a review of systematic reviews. J Adv Nurs. 2004;45(2):117-25.
